# Biomaterials for enhanced immunotherapy

**DOI:** 10.1063/5.0125692

**Published:** 2022-12-16

**Authors:** Nicholas Cunningham, Réjean Lapointe, Sophie Lerouge

**Affiliations:** 1 Research Center, Centre Hospitalier de l'Université de Montréal (CRCHUM), Montreal, Quebec H2X 0A9, Canada; 2 Institut du Cancer de Montréal (ICM), Montreal, Quebec H2X 0A9, Canada; 3 Department of Mechanical Engineering, Ecole de Technologie Supérieure (ÉTS), Montreal, Quebec H3C 1K3, Canada

## Abstract

Cancer immunotherapies have revolutionized the treatment of numerous cancers, with exciting results often superior to conventional treatments, such as surgery and chemotherapy. Despite this success, limitations such as limited treatment persistence and toxic side effects remain to be addressed to further improve treatment efficacy. Biomaterials offer numerous advantages in the concentration, localization and controlled release of drugs, cancer antigens, and immune cells in order to improve the efficacy of these immunotherapies. This review summarizes and highlights the most recent advances in the use of biomaterials for immunotherapies including drug delivery and cancer vaccines, with a particular focus on biomaterials for immune cell delivery.

NOMENCLATURE

ACT: Adoptive cell therapy
APC: Antigen-presenting cells
BSA: Bovine serum albumin
CAR: Chimeric antigen receptor
CDN: Cyclic dinucleotides
CpG ODN: (Cytosine–guanine) oligonucleotides
CTLA-4: Cytotoxic T lymphocyte antigen-4
DC: Dendritic cells
GM–CSF: Granulocyte–macrophage colony-stimulating factor
ICI: Immune checkpoint inhibitors
IL-2: Interleukin 2
MHC: Major histocompatibility complex
MSR: Mesoporous silica rod
MSM: Mesoporous silica microspheres
NK: Natural killer cells
PBMC: Peripheral blood mononuclear cells
PCL: Polycaprolactone
PD-1: Programmed cell death protein 1
PD-L1: Programmed death-ligand 1
PEI: Polyethyleneimine
PIC: Polyisocyanopeptide
PLGA: Poly (lactide-co-glycolide) acid
ROS: Reactive oxygen species
STING: Stimulator of interferon genes
TIL: Tumor-infiltrating lymphocytes
TLS: Tertiary lymphoid structure
T-VEC: Talimogene laherparepvec

## I. INTRODUCTION

Despite continuous progress in detection and treatment, cancer remains one of the leading causes of death worldwide.^1^ Cancer immunotherapy—where cancer treatment is achieved by harnessing and assisting patients' own immune systems—has revolutionized oncology, allowing previously impossible precision in targeting tumor cells compared to conventional treatments and showing impressive results in the previously untreatable disease.^2^ Immunotherapy exploits the inherent response of the host immune system, which can detect the foreign antigens created by cancerous cells and recognize and eliminate malignant cells.

**FIG. 1.**
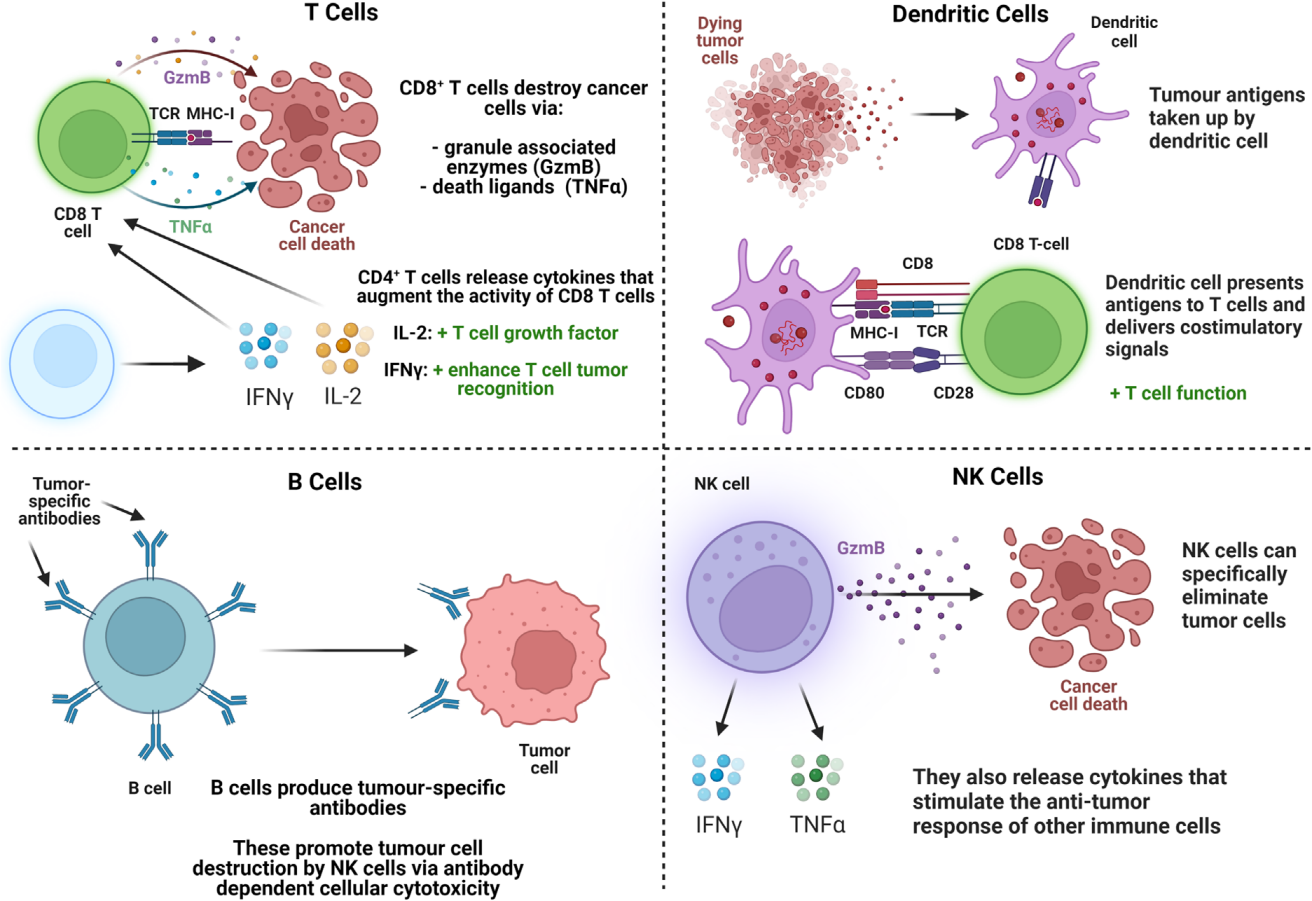
Summary of the main immune cell types relevant to cancer immunotherapy and their functions. GzmB: granzyme B, TNFα: tumor necrosis factor α, IL-2: interleukin-2, IFNγ: interferon γ, TCR: T cell receptor, MHC: major histocompatibility complex.

Cell-mediated immunity is the most relevant part of the immune system in the response to cancer, which in innate immunity consists of polymorphonuclear cells (neutrophils, eosinophils, basophils, and mast cells), phagocytic cells [monocytes, macrophages, and dendritic cells (DCs)], and natural killer (NK) cells. B and T lymphocytes (commonly referred to as B and T cells)—of which there are multiple subtypes—are the primary immune cells involved in adaptive immunity. Immunotherapy relies on the interaction of cells, such as T cells, B cells, DC, and NK cells, with cancers^3–7^ briefly explained in Fig. 1. This process has been described as the cancer immunity cycle, where antigens from necrotic or apoptotic cancer cells are captured by antigen-presenting cells (APCs). The APC subsequently stimulate cytotoxic T lymphocytes to specifically target and destroy the associated tumor cells via apoptosis.^8^ Unfortunately, alone, its efficacy is often limited by the inhibitory effect of the tumor microenvironment and hence the evasion of the immune system by cancer cells.

**FIG. 2.**
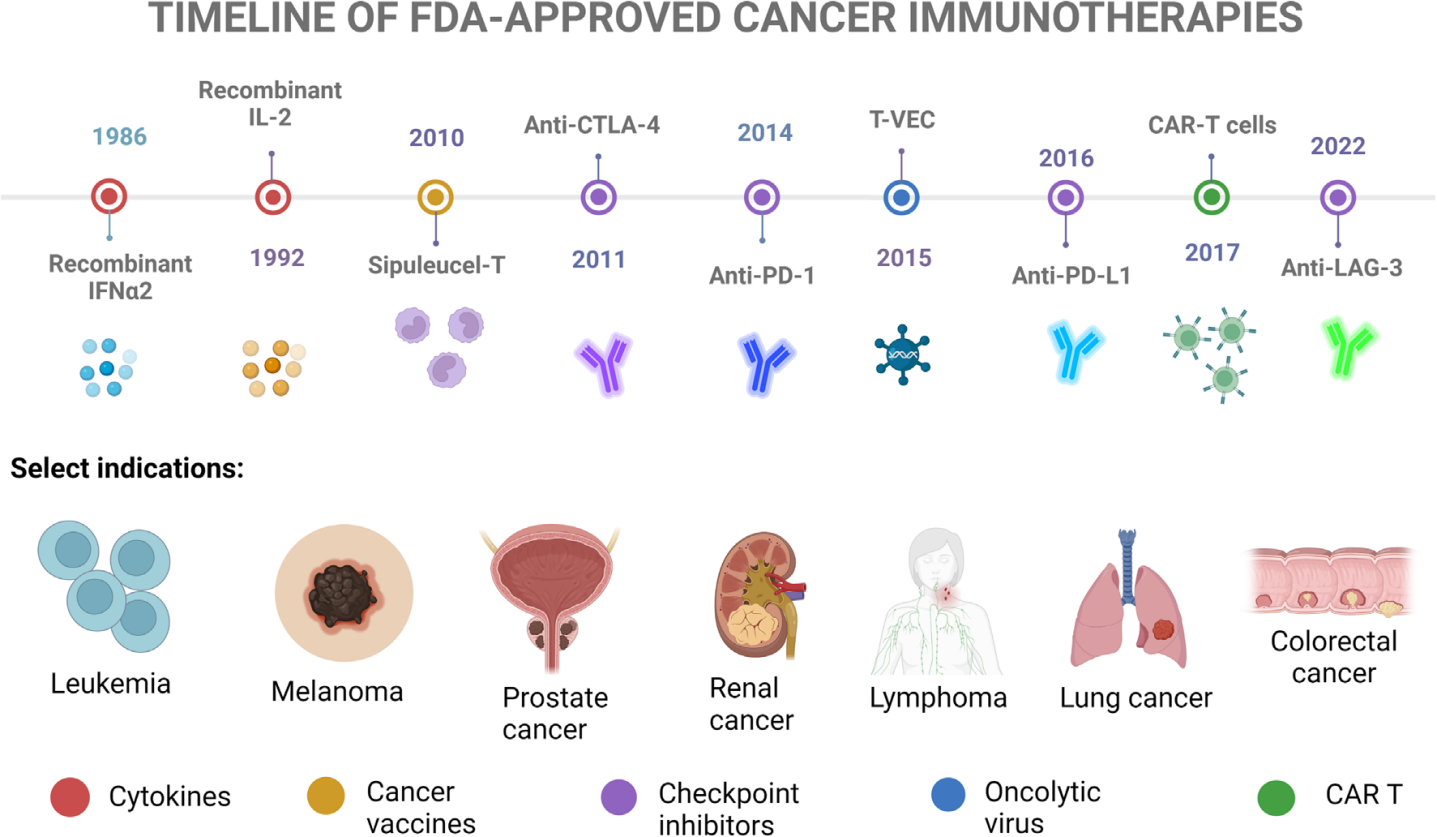
Timeline of selected FDA-approved cancer immunotherapies. Adapted with permission from Cancer Research Institute, see https://www.cancerresearch.org/fda-approval-timeline-of-active-immunotherapies for “FDA Approval Timeline of Active Immunotherapies-Cancer Research Institute 2022.”^16^

Numerous immunotherapies have been developed based on this principle of stimulating and augmenting the immune system. Some are based on drugs enhancing the T cell response, such as Interleukin 2 (IL-2), or immunomodulatory drugs targeting immune checkpoint inhibitors (ICIs). Some treatments encourage the development of immune cells specialized against the tumor (so-called cancer vaccines^9^). Oncolytic viruses (OVs) can both directly destroy cancer cells and produce immunostimulatory cytokines.^10^ Others consist of the injection of cytotoxic T cells, referred to as adoptive cell therapy (ACT).^11^ There are countless ongoing clinical trials, of which it is not possible (and not the aim of this review) to summarize here. Selected FDA-approved immunotherapies are highlighted in Fig. 2 and are explained in detail where relevant in Sec. II.

Despite promising results and numerous clinical studies, the efficacy of these strategies is still limited due to diverse factors, which will be summarized later. A current trend is to combine multiple therapies, particularly a combination of anti-programmed cell death protein 1 (PD-1) and either anti-cytotoxic T lymphocyte antigen-4 (CTLA-4)^12^ or the newly approved ICI targeting lymphocyte-activation gene 3 (LAG-3),^13^ or the combination of these checkpoint inhibitors with ACT for melanoma and ovarian cancers.^14,15^ These combinations are a major focus for current clinical trials with the goal of establishing best practice, and it seems likely that future gold-standard treatments will comprise multiple immunotherapies, perhaps alongside conventional treatments, as combination therapies. Another trend is the increasing use of biomaterials to improve the efficiency of immunotherapies and decrease their toxic effects. This review will focus on the potential of biomaterials (as cell scaffolds or controlled delivery systems for antibodies or tumor antigens) to further enhance the efficacy and decrease the toxicity of immunotherapy.

## II. BIOMATERIALS IN IMMUNOTHERAPY

### A. Introduction and rationale

**FIG. 3.**
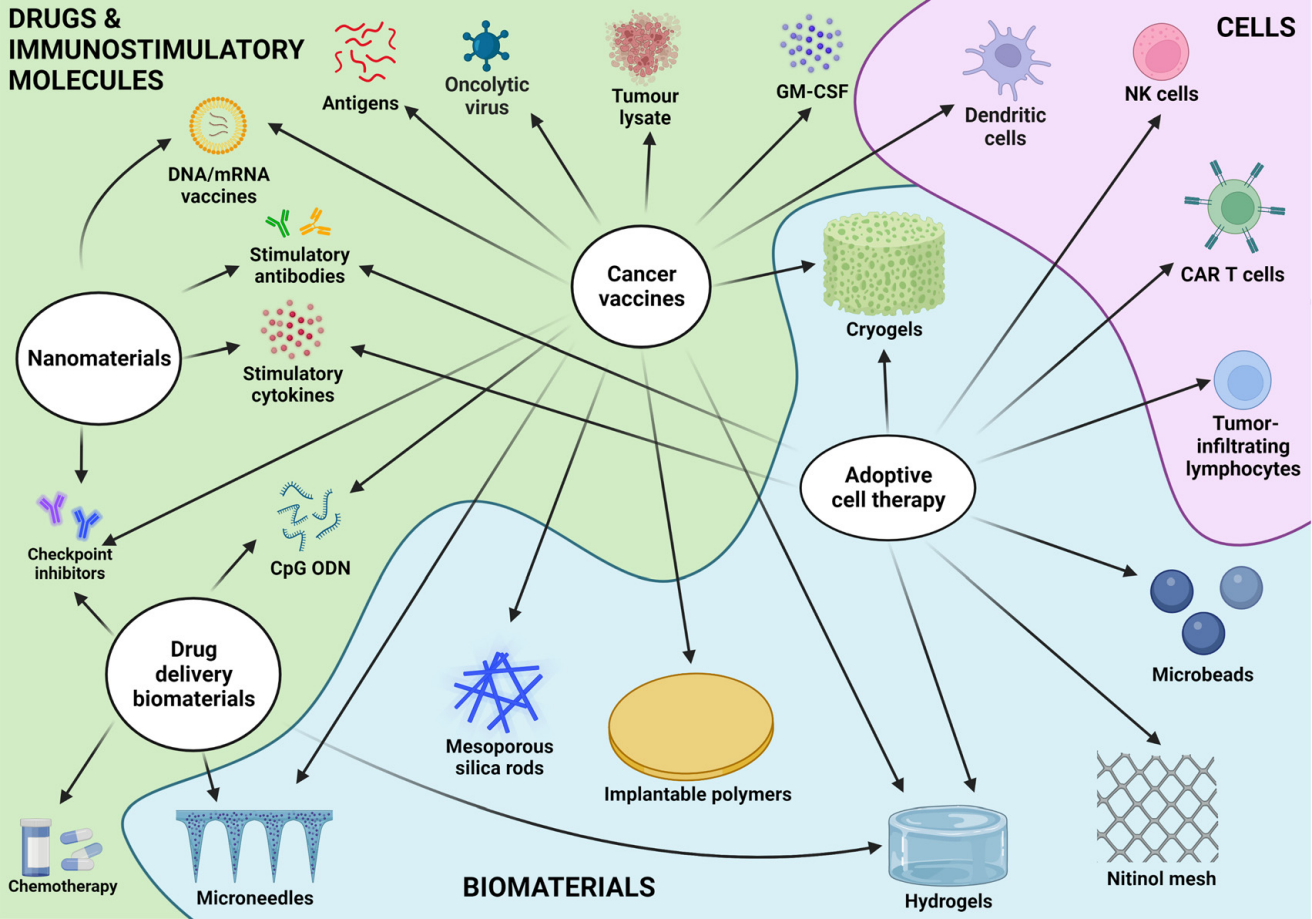
Summary of immunotherapies and the potential role of biomaterials.

Biomaterials, defined as “materials designed to take a form that can direct, through interactions with living systems, the course of any therapeutic or diagnostic procedure”^17^ have long been widely studied in numerous biomedical applications.^18^ We can differentiate biomaterials by their source (natural or synthetic), their class (metals, ceramics, or polymers), and their stability (permanent or biodegradable). Biomaterials have been used in several oncology treatments, for example, for local administration of chemotherapy in urothelial carcinoma^19^ or the embolization of blood vessels for palliative treatment of hepatic cancers,^20^ to name just a few. In the present review, we will limit ourselves to immunotherapies. While only comparatively recently have developments in immunotherapy supported their use in oncology, biomaterials offer numerous possibilities to augment the efficacy of immunotherapies or limit their harmful side effects, as summarized in Fig. 3. In brief, they can achieve this through controlled spatial and temporal release of the cells and immunotherapeutic agents, which can result in dose-dependent and off-target toxicities in current immunotherapies.^21^ They can also create a “niche” for the activation of endogenous or exogenous APC, with potential additional anti-tumor efficacy coming from the biomaterial itself, such as their pro-inflammatory effects or reactive oxygen species (ROS) generation.^22^

Due to the interlinked nature of the immune system, there is extensive crossover in the components and mechanisms of action of immunotherapeutic biomaterials. However, two broad categories that can be proposed are cell-delivery biomaterials, directly incorporating cells such as lymphocytes and DC as localized immunotherapies,^23^ and cell-free biomaterials, which can incorporate a combination of immune adjuvants, antigens and even additional cancer therapeutics such as chemotherapeutic agents alongside the inherent immunomodulatory effects of certain biomaterials.^22,24^ This review will focus on biomaterials in these categories with a particular focus on lymphocytes as antitumoral agents, either through direct administration and stimulation of lymphocytes or by their indirect stimulation by other cells or immunomodulatory components.

### B. Cell-free biomaterials

#### 1. Aim and design criteria

The aim of cell-free scaffolds for immunotherapies is to provide, either through their own chemical composition or through attached or encapsulated biological factors, cellular cues to direct and encourage a favorable immune response toward a given stimulus, or alternatively to locally deliver anti-cancer drugs. In the case of a biomaterial for cancer immunotherapy, this would implicate either the inclusion of immune agonists and/or immunotherapeutic drugs, within a material matrix suitable for the administration and controlled delivery of these factors, such that a strong anti-cancer response is achieved, and/or the utilization of the inherent immune response to a particular matrix to recruit and activate APC.

These systems should be composed of a biocompatible material, which according to the definition of biocompatibility must not elicit adverse biological effects (cytotoxicity, carcinogenicity, hypersensitivity, etc.), but should promote an “appropriate host response in a specific application,”^17^ namely, here help recruit and activate the APC or be at least immunologically inert to allow the function of its immunomodulatory components. Indeed, in this particular case detailed here, provoking some immune response is deemed beneficial in the anti-cancer response and some inflammation could even be encouraged.

Such a material should also be:

• Injectable through a small needle to enable minimally invasive procedures.
• Persistent over the necessary timescale for maximum efficacy of its drug or molecule of choice and ideally be degraded afterwards.
• For drug delivery, the release rate must be well controlled by the scaffold properties and degradation rate.
• The use of simple materials, potentially already used in FDA-approved medical devices or therapies, is generally desired for easier regulatory approval.

Sections II B 2–II B 5 detail the principles and review the cases of biomaterials used for cancer vaccines and controlled release of OV or immunotherapeutic drugs.

#### 2. Biomaterials for cancer vaccines

**FIG. 4.**
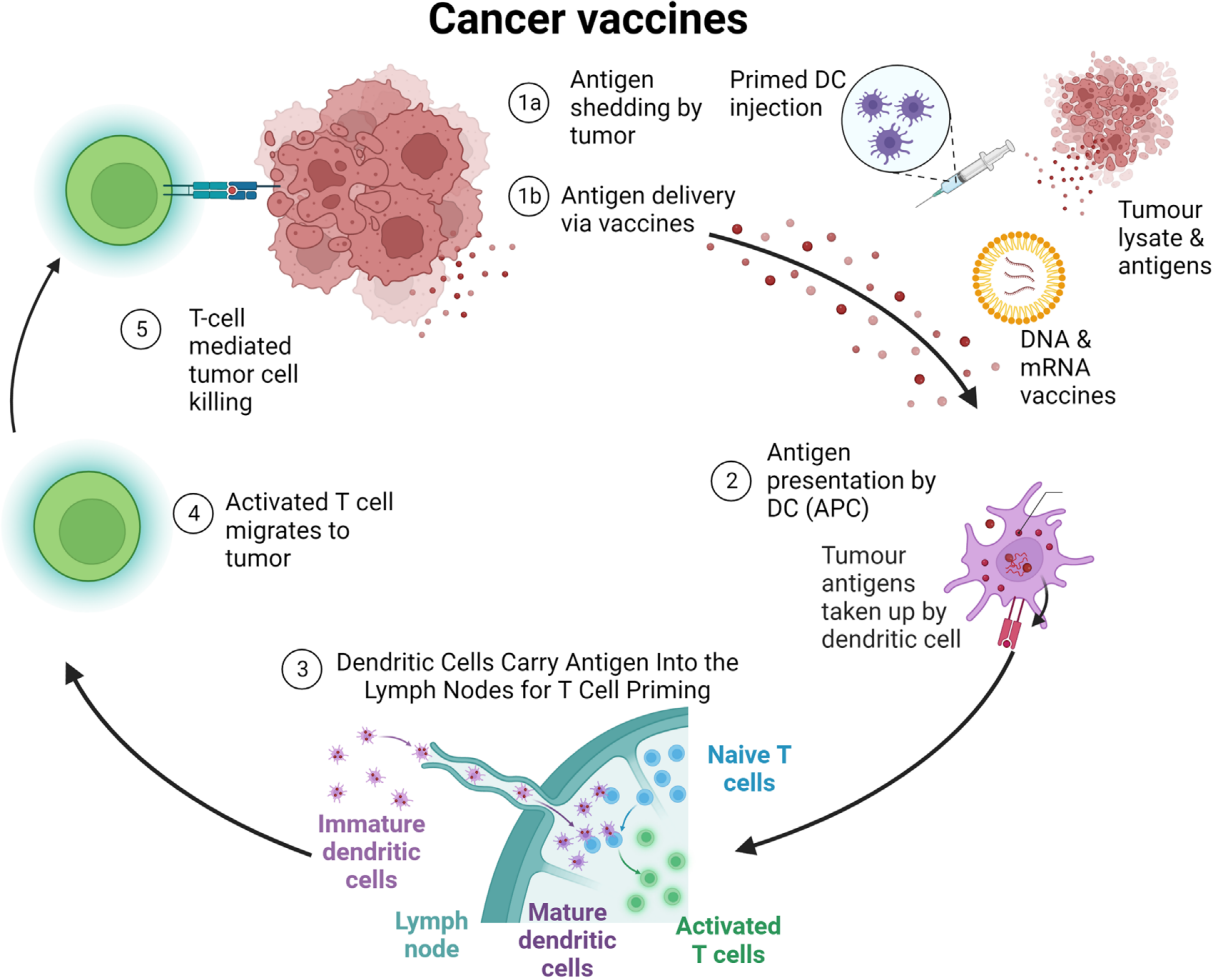
Cancer vaccine mechanisms. Tumor antigens from tumor cell death or administered vaccines are taken up by dendritic cells for priming of tumor-specific T cells, which can then specifically eliminate cancer cells, continuing the cycle.

Cancer vaccines can include dead tumor cells or lysate, DC, antigens, or nucleic acids, such as mRNA, with DC the most widely studied form of cancer vaccine to date.^25^ Whatever their component, the vaccines—generally administered subcutaneously, intramuscularly, or intravenously—act to supplement or improve tumor-specific lymphocyte activity via improved antigen presentation, lymphocyte activation, and localization of the immune response to the tumor.^9^ A summary of cancer vaccine mechanisms is shown in Fig. 4.

Exogenous cells can be added to the antigens injected, to ensure the formation of activated DC. This is the case of Sipuleucel-T (Provenge^®^), the first immune cell therapy approved by the FDA as a cancer treatment.^26^ Sipuleucel T improved antitumor CD8^+^ cell response and survival in a clinical trial in patients with castration-resistant prostate cancer.^27^ Despite this initial success, limited progress has since been made in the clinical use of cancer vaccines, with other candidate vaccines failing to demonstrate clinical efficacy and leaving Sipuleucel-T as still the only FDA-approved cancer vaccine.^28^ Nevertheless, research continues with recent developments, such as neoantigen vaccines, which target patient-specific antigens rather than tumor-associated antigens that are common between patients and associated with higher immune tolerance.

To be effective, cancer vaccines must have two main properties. First, they must stimulate the appropriate specific immune responses against the correct target. Second, the immune responses must be powerful enough to overcome the barriers that cancer cells use to protect themselves. Therefore, sustained delivery (to avoid rapid clearance) and an appropriate structure for the APC to interface with the vaccine component is key.

**FIG. 5.**
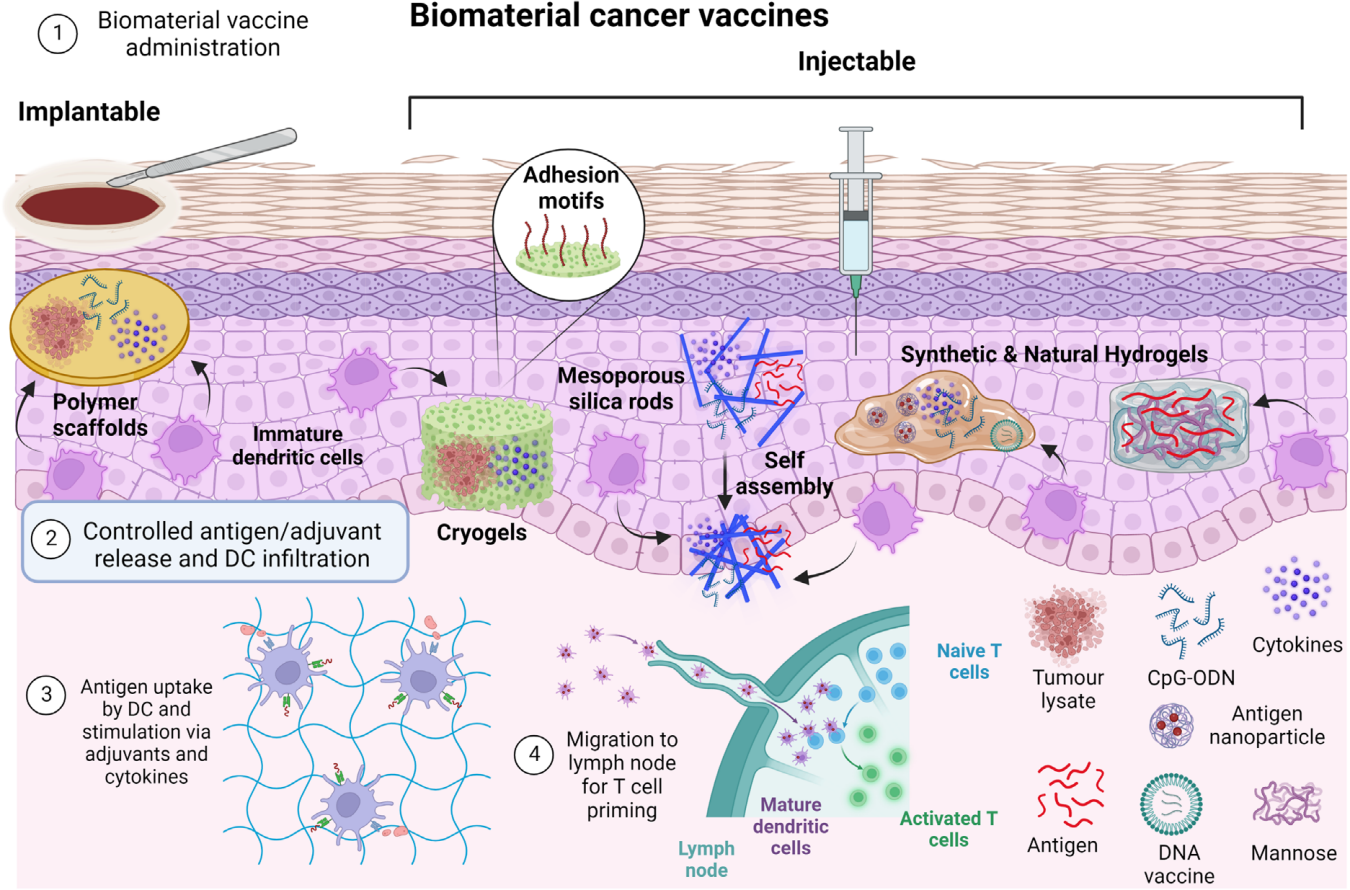
Principles of biomaterial vaccines. Biomaterials containing tumor lysates, antigens, cytokines, and adjuvants are implanted or injected. Antigens and adjuvants are released in a controlled manner and DC infiltrate the biomaterial niche where antigen uptake can occur. DC then migrate to lymph nodes where T cell priming occurs.

Biomaterials have thus been used to create a physical structure loaded with vaccine components which stimulate APC cells *in situ*. These will then disperse to lymph nodes and activate resident T cells which then travel to the tumor site and eliminate malignant cells. We can distinguish between implantable and injectable scaffolds. Figure 5 describes the two main approaches, while Table I summarizes the main biomaterials used for the different vaccines.

**TABLE I.** Biomaterials used for cancer vaccines.

Materials	Therapeutic agents	Injectable?	Immunotherapy model and outcomes	References
Poly (lactide-co-glycolide) acid (PLGA)	Tumor lysate antigens, GM-CSF, (cytosine-guanine) oligonucleotides (CpG ODN), PD-1 or CTLA-4, monophosphoryl lipid A, polyinosinic:polycytidylic acid	No	Mouse melanoma models	29–31
			Up to 90% survival increase with vaccine + GM-CSF + CpG ODN	
			Enhanced effect in combination with PD-1 or CTLA-4	
Methacrylated alginate (MA-alginate)	Tumor lysate antigens, GM–CSF, (cytosine–guanine) oligonucleotides (CpG ODN)	Yes	Mouse melanoma models	32, 33
			Up to 80% survival with biomaterial vaccine + GM–CSF + CpG ODN vs 60% with bolus vaccine	
MA–alginate + methacrylated polyethylene glycol (MA–PEG)	Tumor lysate antigens, GM–CSF, (cytosine–guanine) oligonucleotides (CpG ODN)	Yes	Mouse leukemia models	34
			100% survival with chemotherapy + biomaterial vaccine	
Alginate + tumor lysate	Tumor lysate antigens, GM–CSF, anti-PD-1	No	Mouse pancreatic cancer model	35
			Five-fold decrease in tumor volume compared to treatment with PBS, additive effect with anti-PD-1	
Mesoporous silica rods (MSR)	Tumor antigen, GM–CSF, anti-PD-1, polyethyleneimine (PEI)	Yes	Mouse lymphoma, melanoma, and lung cancer models	36–38
			Increased DC recruitment and survival with MSR vaccines	
PCL–PEG–PCL	GM–CSF, OVA nanoparticles	Yes	Improved DC recruitment *in vivo*, increased T cell response to EG7-OVA *in vitro*	39
Bovine serum albumin (BSA) + PCL–PEG–PCL	DNA vaccine	Yes	Improved DC recruitment and increased T cell response to amyloid-β Alzheimer antigen *in vivo*	40
CpG-modified carboxymethyl chitosan and partially oxidized mannan	OVA antigen	Yes	Mouse melanoma model	41
			Reduced tumor growth and improved survival with gel vaccine	
Catechol chitosan + calcium phosphate nanosheet	OVA antigen	Yes	Increased DC activation and antigen presentation	42

Biomaterial vaccines were among the first biomaterial-delivered immunotherapies to be researched, with pioneering work from Mooney's group being essential in this field and influential in its development.

The first development was an implantable PLGA scaffold incorporating tumor lysate antigens (from destroyed cancer cells), GM–CSF as a stimulatory cytokine for DC and (cytosine–guanine) oligonucleotides (CpG ODN) a toll-like receptor 9 (TLR-9) agonist that also activates DC.^29^ The interconnected pores in the scaffold create an environment in which infiltrating DC are activated and process the tumor lysate antigens before migrating to lymph nodes where they can prime antigen-specific T cells. The scaffold also regulates the release of antigens and adjuvants to encourage a persistent immune response. This biomaterial-based vaccine paved the way for future developments in biomaterial immunotherapies and has the distinction of being the first such biomaterial to enter clinical trials, where the above vaccine is being tested in a phase I clinical trial to determine its safety and feasibility and to confirm its biological activity when used to treat metastatic melanoma.^43^ The study completion date is in 2022. The group has further developed the scaffold, showing that the vaccine is even more effective in combination with checkpoint inhibitors.^30^ Furthermore, alternative vaccine adjuvants, such as monophosphoryl lipid A and polyinosinic:polycytidylic acid, also preventively and therapeutically reduced tumor growth in mouse melanoma models.^31^

Later, the team developed an MA–alginate “cryogel” scaffold functionalized with RGD binding sites and using the same immunomodulatory factors, which demonstrated both therapeutic and preventative effects in mice melanoma models, with a further benefit of injectability as opposed to the previous PLGA scaffold.^32^ However, this requires quite large diameter needles (16G). According to *in vitro* tests, approximately 80% of the encapsulated vaccine compounds (GM–CSF and CpG ODN) were released within the first 4 days, followed by slow and sustained release over the next month. The interconnected macroporous structure allowed cellular infiltration and immune cell trafficking *in situ.* Further work interestingly showed that the vaccine maintained its efficacy regardless of whether the injection site was adjacent or distal to the tumor and draining lymph nodes.^33^ This group also developed a similar cryogel using a combination of MA–alginate and PEG to eradicate established acute myeloid leukemia in mice.^34^ Lu *et al.* developed a relatively similar cryogel, which incorporates tumor cell lysates during hydrogel preparation and significantly reduced the growth of a secondary tumor after surgical resection of the primary tumor.^35^

All these 3D biomaterials are, however, fabricated *ex vivo* and require either surgical placement in the body or large invasive needles for implantation. Moreover, they are not biodegradable and their preformed structures could limit the capability of host cells to organize themselves.^36^

To solve this issue, Mooney's group proposed self-assembling mesoporous silica rods (MSR) that are more easily injectable (18G needle), biodegradable, and create more macroporous 3D structures for better interaction with immune cells. The MSRs are injected with a needle and spontaneously assemble *in vivo*, degrading over time and allowing a slow release of vaccine antigens and adjuvants.^36^ These self-assembled MSR scaffolds increased the number of recruited cells compared to previously reported preformed macro-porous polymer scaffolds, with 20 × 10^6^ cells in the MSR vaccine after 5 days *in vivo* vs only 6 × 10^6^ in the MA–alginate cryogel vaccine after 6 days. In later work, the MSR vaccine was coated with polyethyleneimine (PEI) to enhance the effect of neoantigen peptides.^37^ MSR–PEI vaccines significantly enriched the DC population, roughly doubling the number of recruited DC, and enhanced host DC activation and T cell responses compared to the existing MSR vaccine. The MSR vaccines eradicated established E7-ovalbumin (OVA) tumors in 80% of mice and showed efficacy in reducing tumor growth in melanoma and carcinoma cell lines, with an additive effect when combined with CTLA-4 injection, demonstrating the potential of biomaterials-based vaccines to function alongside other immunotherapies such as checkpoint inhibitors.^37^ Further work using MSR vaccines has applied the same principle for alternative peptide antigens, such as gonadotropin-releasing hormone (GnRH) and HER2/neu, implicated in multiple cancers including breast cancer.^38^

Other groups have developed biomaterial vaccines with similar stimulatory molecules, using other commonly used biomaterials such as chitosan, polycaprolactone (PCL), and even bovine serum albumin (BSA). For example, Sun *et al.* developed an injectable thermosensitive PCL–PEG–PCL hydrogel encapsulated GM–CSF and chitosan-coated ovalbumin (OVA) nanoparticles to create the immune cell niche for DC activation,^39^ where the chitosan itself may induce DC maturation via the Stimulator of Interferon Genes (STING) signaling pathway.^44^ STING is a transmembrane protein that interacts with cyclic dinucleotides (CDNs) produced in response to cytosolic double-stranded DNA, resulting in the production of IFN-β and other cytokines that promote the *in vivo* anti-tumor T cell response.^45^ In another study by Giang Phan *et al.*, BSA was crosslinked with a similar PCL–PEG–PCL hydrogel to deliver a DNA vaccine against the amyloid-β Alzheimer antigen.^40^ Both PCL–PEG–PCL hydrogel vaccines induced strong immune responses and improved DC recruitment. Note that hydrogels are particularly interesting as they often present shear-thinning behavior. Moreover, some can undergo reversible volume phase transitions or sol-gel phase transitions in response to external physical or chemical stimuli—such as temperature, pH, ionic strength, light, and electromagnetic radiation—called stimuli-sensitive or intelligent hydrogels and allowing *in situ* gelation and hence minimally invasive treatment.

Chitosan seems to be gaining popularity for use in cancer vaccines, with an injectable gel formed by a Schiff base reaction between CpG-modified carboxymethyl chitosan and partially oxidized mannan increasing DC infiltration and maturation and significantly reducing tumor growth in a B16-F10 mouse melanoma model.^41^ Furthermore, though not tested in tumor models, an OVA-antigen-containing “nanosheet” was formed through the simple mixing of catechol-modified chitosan, PBS, and CaCl_2_.^42^ The nanosheet significantly increased DC activation and antigen presentation compared to free antigen and so could be another promising future carrier of antigens as a cancer vaccine. Carroll *et al.* showed that chitosan, a cationic polymer, can engage the cGAS-STING pathway to mediate the selective production of type I IFN and interferon-stimulated genes, which were then responsible for mediating the activation of DC and induction of cellular immunity.^44^ More generally, these studies show that the choice of the biomaterial composition and porosity of the scaffold is key to induce successful recruitment and activation of DC. Moreover, hydrogels have the advantage of being injectable and can play the dual role of being a vaccine carrier with sustained release and a platform for recruiting DCs.

While clinical results are still absent, all these results suggest that biomaterials will play a key role in the development of cancer vaccines and that, alongside other immunotherapies, vaccines will have a role to play in therapeutic cancer treatment. Furthermore, while still at an early stage, the prophylactic vaccination of cancers with known antigens, such as human epidermal growth factor receptor-2-positive breast cancers, is an intriguing possibility.^46^ Prophylactic vaccination could be targeted to genetically high-risk populations, or alternatively used to prevent cancer recurrence and metastases.^47^ Cancer vaccines may also be pioneering precursor technologies, demonstrating the benefit of antigens, adjuvants, and DC, which can then be included as additional components of other immunotherapies.

#### 3. Oncolytic viruses

**FIG. 6.**
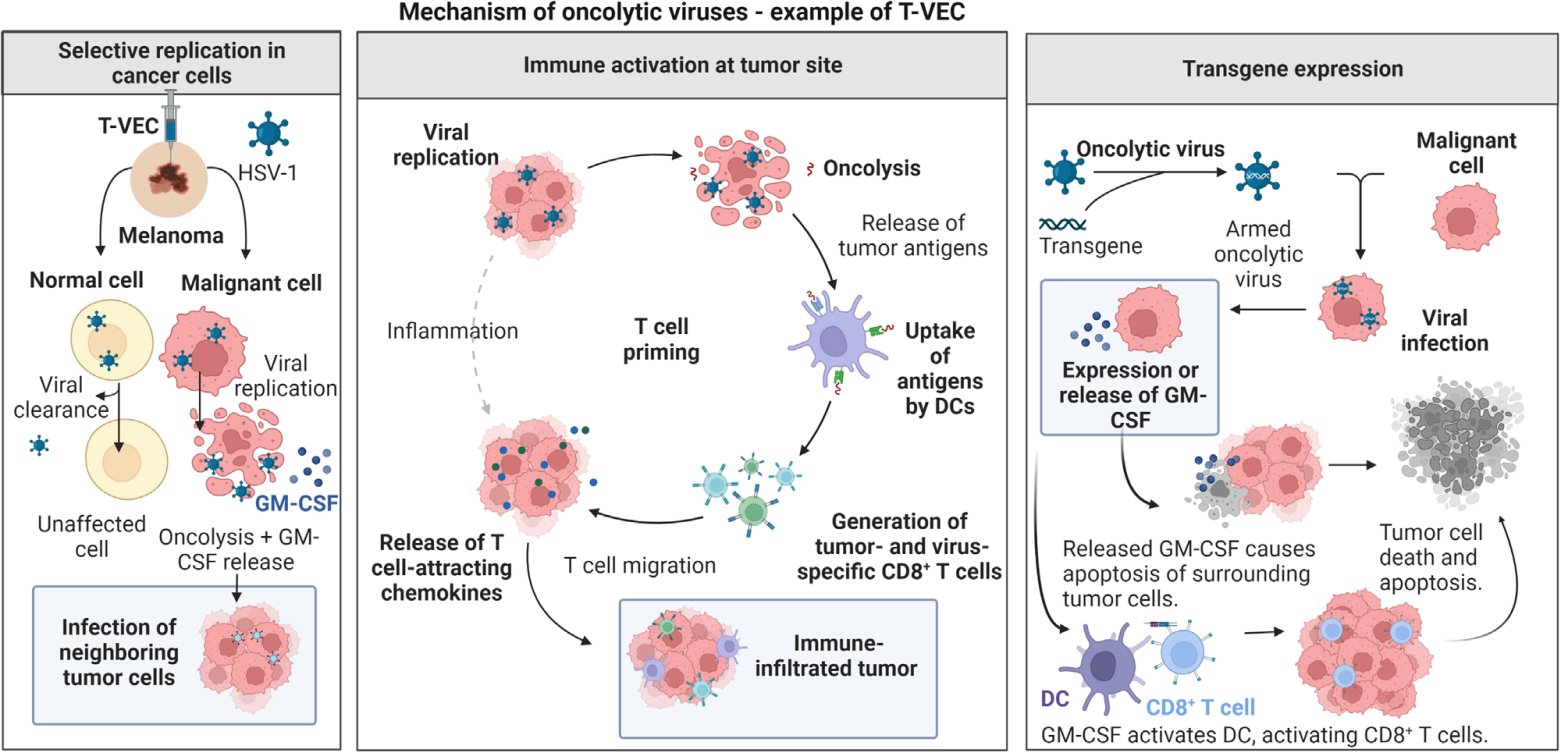
Oncolytic virus mechanisms: Oncolytic viruses such as T-VEC are selectively replicated in cancer cells, eliminating tumor cells via oncolysis and stimulating the immune system via the antigen release of destroyed tumor cells and expressed transgene products such as GM–CSF.

OV are genetically modified to infect and lyse only cancer cells that sacrifice their normal antiviral defenses in order to grow more rapidly.^10^ OV can, thus, selectively infect and kill cancer cells, while leaving surrounding non-cancerous cells unharmed. While the precise mechanisms of their anti-tumor effect remain to be defined, they include direct tumor cell lysis, recruitment of APC, and tumor infiltrating lymphocytes as well as the release of pro-inflammatory cytokines from lysed tumor cells (Fig. 6). In addition, some OV have been genetically modified to express these cytokines in order to augment their anti-tumor activity.^48^ They are a somewhat niche area of cancer immunotherapy, with only one OV to date approved by the FDA: talimogene laherparepvec (T-VEC), a herpes simplex virus that showed a significant clinical response in the treatment of unresectable melanoma, with the virus modified to also express granulocyte–macrophage colony-stimulating factor (GM–CSF).^49^ Recent work has proposed the use of biomaterials in the form of nanofibers^50^ or nanoparticles^51^ for targeted delivery of OV, with bulk biomaterials for OV delivery summarized in Table II and subsequently explained further.

**TABLE II.** Bulk biomaterials for oncolytic virus delivery.

Materials	Therapeutic agents	Injectable?	Immunotherapy model and outcomes	References
Alginate	Oncolytic adenovirus	Yes	Mouse xenografts of human cervical cancer and glioma	52
			Increased OV accumulation in the tumor, reduced tumor growth, and reduced OV accumulation in off-target tissues	
Gelatin-hydroxyphenyl propionic acid hydrogel	Oncolytic adenovirus	Yes	Hamster models with pancreatic carcinoma	53
			Reduced tumor growth, reduced OV accumulation in off-target tissues, reduced anti-OV immune response	
Silk elastin-like protein polymer hydrogel	Oncolytic adenovirus	Yes	Mouse xenograft of human head and neck cancer	54
			Reduced tumor growth and greater OV persistence at the tumor site	
Polyurethane-sulfamethazine hydrogel	Oncolytic adenovirus	Yes	Mouse xenograft of human lung cancer	55
			Reduced tumor growth and greater OV persistence at the tumor site	

Choi *et al.* used an alginate gel to encapsulate OV which was shown to double their accumulation in the tumor and reduced tumor growth by half compared to non-encapsulated OV in mouse xenograft models of human C33A (cervical) and U343 (glioma) cancer cell lines. Gel encapsulation also reduced OV accumulation in off target tissues.^52^ Work from Yun’s group showed similar improvements in OV efficacy, with a gelatin-hydroxyphenyl propionic acid (GHPA) hydrogel also showing a twofold increase in anti-tumor efficacy, reduced accumulation in off target tissues, and a reduced anti-OV immune response in hamster models with the HaP-T1 hamster pancreatic carcinoma cell line.^53^ In murine xenograft models of localized OV delivery, both a recombinant silk elastin-like hydrogel in a head and neck cancer model and a polyurethane-sulfamethazine injectable hydrogel in a lung cancer model also reduced tumor growth by around half compared to non-encapsulated OV. Histological analysis also demonstrated greater persistence of the OV at the tumor site in these cases.^54,55^

Clearly, biomaterials have the potential to improve OV retention in tumors, protect them from elimination by the immune system, and hence improve their efficacy. Clinical trials of locally delivered OV will no doubt be awaited with interest as this immunotherapy experiences a resurgence.

#### 4. Drug delivery biomaterials

Biomaterials for simple local delivery of anti-cancer drugs have also been investigated, as with numerous other medical domains.^56^ Drug delivery scaffolds and the principles underlying the design of hydrogel drug delivery systems, focusing on the physical and chemical properties of the hydrogel network and the hydrogel–drug interactions, have been reviewed previously and will not be detailed here.^57–61^ Biomaterial scaffolds allow local and controlled release of drugs and molecules, therefore allowing lower doses for equivalent efficacy and limiting dose-related toxicities. Spatial control also has the double benefit of increasing the payload of the drug or molecule at the relevant site, while ensuring a minimum is lost to non-tumor sites where it would be ineffective or even potentially cause off-target toxicity.

Whatever drugs and molecules are being delivered, the drug dosage, mechanism of release, and ease of administration of the biomaterial are of greater importance. Here, the ability to chemically conjugate molecules of interest to the material, and easier administration, such as with an injectable material, would be highly desirable.

**FIG. 7.**
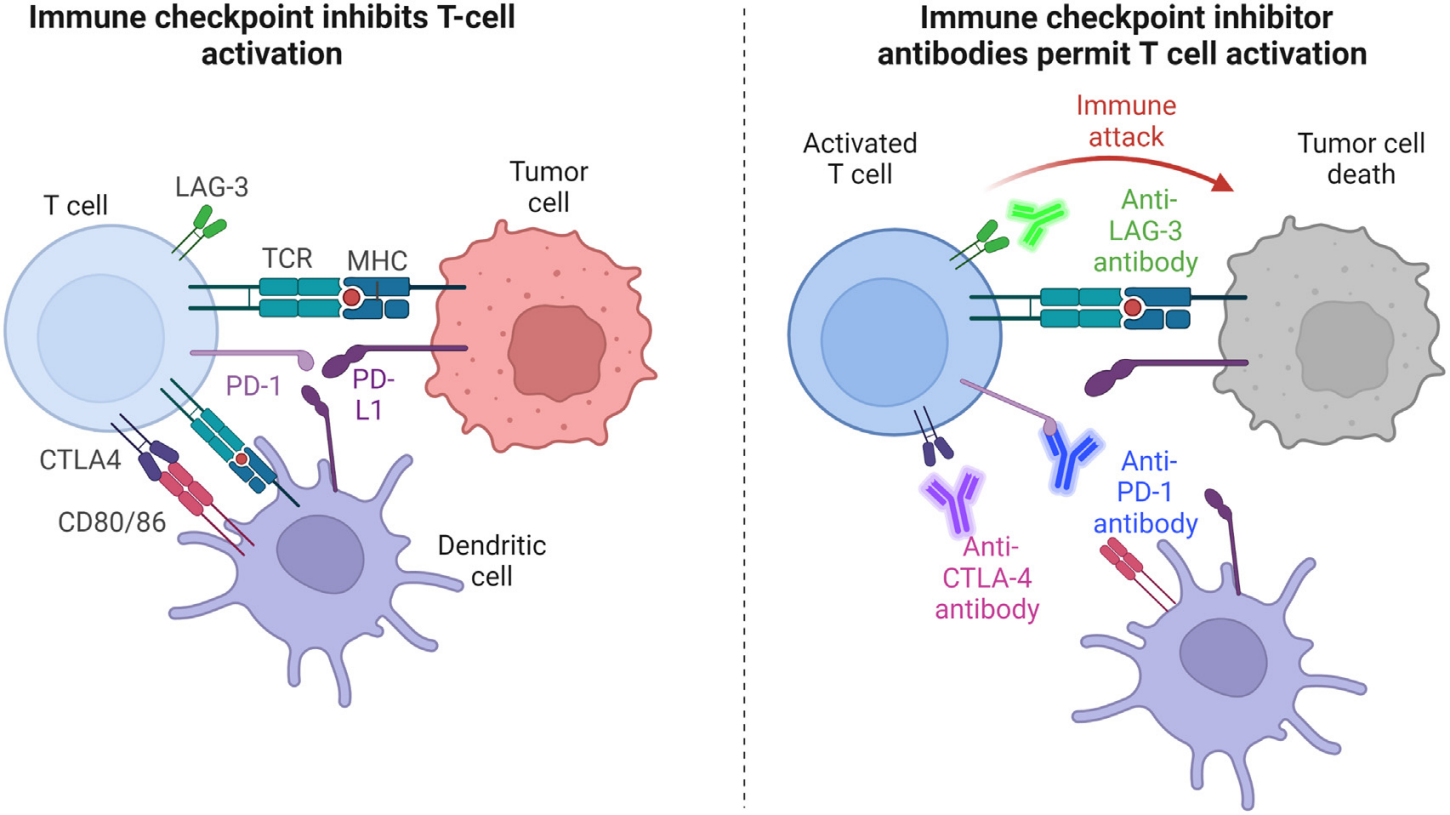
Mechanism of checkpoint blockade: antibodies inhibit binding of immune checkpoint pathway receptors, such as PD-1, CTLA-4, and LAG-3, preventing T cell inhibition and hence allowing tumor attack by activated T cells.

In terms of immunotherapeutic drugs, immune checkpoint inhibitors (ICIs) are among the most popular for local drug delivery, often combined with other drugs. ICI are monoclonal antibodies targeted toward so-called “immune checkpoints”—inhibitory immune pathways that downregulate T cell activation when their receptors on immune cells are activated that aim to maintain organism self-tolerance and avoid autoimmunity. Cancer cells themselves can develop the ability to activate these receptors and hence escape the downregulated immune system, for example, programmed death-ligand 1 (PD-L1) expressed on tumor cells (see Fig. 7). The most commonly targeted ICI are CTLA-4, which competes with CD28 to bind CD80/CD86 and hence downregulates T cells,^62^ PD-1, which has an inhibitory effect on T cell activation while stimulating Treg cells, which suppress the anti-tumoral immune response, and the ligand of PD-1, PD-L1, which can be found on the surface of tumor cells as well as endogenous epithelial and immune cells and contributes to the downregulating of T cells in the tumor microenvironment. LAG-3, a new addition to the ICI portfolio, is a cell surface protein expressed on immune cells that downregulates T cell proliferation and function.^63^

ICI have become one of the most successful immunotherapies and dominate among FDA-approved immunotherapies and immunotherapy clinical trials, with Ipilimumab the first commercially approved ICI, targeting CTLA-4. Nivolumab, targeting PD-1, followed. Both treatments demonstrated improved survival over chemotherapeutic agents as treatments for metastatic melanoma patients, especially PD-1 that has the advantage of reduced toxicity compared to CTLA-4.^64^ ICI have had a revolutionary impact in oncology, although durable responses to ICI remain limited for certain cancers, and their associated toxicities remain a limitation to be addressed.^65^

Dosage and localization clearly contribute to the limitations associated with ICI, with ICI autoimmunity resulting from the systemic delivery of the treatment. For this reason, localized delivery of the drugs could result in more widely usable and safer treatments. However, the rapid and variable release of drugs in conventional delivery means that carriers, such as biomaterial scaffolds, are required for the controlled release and sustained delivery of therapeutics.^58^ Selected biomaterials for immunotherapeutic drug delivery are summarized in Table III and explained further below.

**TABLE III.** Biomaterials for immunotherapeutic drug delivery.

Materials	Therapeutic agents	Injectable?	Immunotherapy model and outcomes	References
Alginate	Celecoxib, anti-PD-1	Yes	Mouse melanoma model	66
			Reduced tumor growth and increased mouse survival, enhanced effect with dual-drug administration with hydrogel	
ROS-degradable poly(vinyl alcohol) hydrogel	Gemcitabine, anti-PD-L1	Yes	Mouse melanoma model	67
			Improved mouse survival, ROS-scavenging effects	
Multidomain peptide hydrogel	Anti-PD-1, CDN	Yes	Mouse oral carcinoma model	69, 70
			Fewer tumor lesions in hydrogel-treated mice, improved mouse survival	
Fibrin gel	Doxorubicin (DOX)-loaded platelet-derived extracellular vesicles, anti-PD-L1	No (sprayable)	Mouse melanoma model	71
			Increased mouse survival, decreased tumor growth in local and distal tumors	
Implantable polycarbonate optical fiber	Anti-CTLA-4, anti-PD-1	No	Mouse melanoma and breast cancer models	72
			Reduced tumor growth and increased mouse survival, treatment guided by tumor impedance	
Polyvinyl alcohol + chitosan microneedles	1-methyl-D,L-tryptophan, anti-PD-L1, indocyanine green	No	Mouse melanoma model	73, 74
			Greater anti-PD-L1 retention at the administration site, decreased tumor growth, and greater survival. Improved effect with indocyanine green photosensitizer	

For example, an alginate hydrogel incorporating anti-PD-1 and the anti-inflammatory drug celecoxib reduced tumor growth by 90% compared to a blank hydrogel in a mouse B16-F10 melanoma model.^66^ The sustained co-delivery of celecoxib and PD-1 enhanced their effects in a synergistic manner, where PD-1 augmented the inhibition of tumor angiogenesis provoked by celecoxib, whereas the anti-inflammatory effect of celecoxib downregulates inflammatory genes which may otherwise interfere with the therapeutic effect of PD-1. Another interesting example consisted of a poly(vinyl alcohol) hydrogel, crosslinked with a compound that can be oxidized and hydrolyzed in the presence of ROS such as H_2_O_2_ in the tumor microenvironment.^67^ This led to gel degradation at the tumor site where ROS are highly prevalent, and the subsequent controlled release of the chemotherapy drug gemcitabine and anti-PD-L1 antibodies. The local release of this drug combination resulted in over 60-day mouse survival in a B16-F10 melanoma model, where no mice in any untreated or single-treatment control groups survived after two months. In addition to controlling the drug release, the hydrogel acts as a ROS scavenger that may limit the ROS-induced differentiation of macrophages to the tumorigenic “M” phenotype.

An example of biomaterials gaining popularity for immunotherapeutic drug delivery is multidomain peptide (MDP) hydrogels. One example developed by Hartgerink's group consists of an amphiphilic core of amino acids, which self-assemble into nanofibers, and gels upon interaction with a negatively charged multivalent.^68^ This hydrogel was used to locally deliver anti-PD-1 in a sustained and controlled manner, which was significantly more effective and durable than systemic anti-PD-1 in preventing oral carcinomas in mice, with 20% of hydrogel-treated mice showing high-grade lesions 5 weeks after treatment compared to 60% of control mice.^69^

The gel was further developed with the addition of CDN as a STING agonist as well as synthesizing another MDP that included an inhibitor of the pro-tumorigenic enzyme inducible nitric oxide synthase.^70^ The cationic gel further slowed and controlled CDN release for longer drug persistence compared to the previous MDP and resulted in the greatest tumor size reduction and survival among treatment groups.

Alternative biomaterial formats for immunotherapeutic drug delivery have also been developed. For example, Zhao *et al.* designed a sprayable fibrin gel containing doxorubicin (DOX)-loaded platelet-derived extracellular vesicles (which preferentially target circulating tumor cells) and anti-PD-L1,^71^ which permitted sustained and controlled release of the two therapies at the tumor site in a murine B16-F10 model. Their synergistic effect greatly decreased tumor growth and increased survival compared to single treatments. Furthermore, tumor growth was also limited in a distal tumor, showing the abscopal effect of the treatment.

In another study, Chin *et al.* developed an implantable optical fiber device, which could simultaneously deliver ICI locally and measure tumor growth in real time by measuring tumor impedance.^72^ The device included photodynamic therapy as a combination therapy, where an administered photosensitizer will create ROS that can both damage tumor cells and vasculature in response to light and improve intratumoral drug retention. Its combination with repeated ICI delivery directed by impedance measurements resulted in a durable anti-tumor response in a range of mouse melanoma and breast cancer models, and though the device is relatively invasive its real-time tumor growth monitoring to direct treatment is advantageous and it could be seen as analogous to routine medical devices such as insulin-delivery pumps.^75^

Another relatively novel biomaterial drug delivery method is the use of microneedles, micrometer sized needles that are associated with faster action, increased efficacy, and better patient compliance compared to transdermal injection.^76^ Microneedle patches, composed of a polyvinyl alcohol core containing the melanoma drug 1-methyl-D,L-tryptophan, surrounded by a chitosan needle shell containing anti-PD-L1 showed improved anti-PD-L1 retention at the administration site as well as decreased tumor growth and greater survival compared to untreated mice and mice with intra-tumor drug delivery using conventional needles.^73^ Later, indocyanine green, a photosensitizer used in photothermal therapy was added to further stimulate the anti-tumor immune response.^74^ These microneedles had an even greater anti-tumor effect and a further survival increase compared to the previous microneedles.

These results demonstrate the still unexplored potential of novel drug delivery systems in immunotherapy, which will only become more necessary as these treatments improve and are approved for other malignancies.

#### 5. Nano-scale biomaterials

While their mechanisms differ from localized bulk biomaterials, nanoparticles have seen interest in both cancer vaccine and tumor drug delivery, due to their ability to act systemically but in a highly targeted manner.^25^ With targets and functions similar to certain bulk biomaterials used for cancer immunotherapy, nanoparticles represent an alternative material form and perhaps an intermediate treatment classified somewhere between systemic therapies and localized biomaterials for cancer immunotherapy. Below are a few examples of the use of nanoparticles in immunotherapy, either as drug or OV delivery systems or means to activate the immune response. Selected nanoparticles used for immunotherapy are summarized in Table IV and explained further below.

**TABLE IV.** Nanoparticles for immunotherapy.

Nanoparticle	Immunotherapeutic agent(s)	Immunotherapy model and outcomes	Reference
Lipid nanoparticle	IL-15 superagonist, IL-21, Pmel-1 T cells	Mouse melanoma model	77
		Complete tumor elimination with cytotoxic T cells + nanoparticles	
PEG–PLGA	PD-1, antitumor necrosis factor receptor superfamily member 4	Mouse melanoma model	78
		Threefold increased survival with dual-drug nanoparticle delivery	
Chitosan	Anti-PD-L1	Mouse melanoma model	79
		Increased anti-PD-L1 retention, mouse survival and fewer metastases	
Iron-dextran + quantum dots	CD28, major histocompatibility complex-peptide complexes, cognate T cells	Mouse melanoma model	80
		Significant tumor growth reduction	
PEGylated oligopeptide-modified poly(β-aminoester)	Oncolytic adenovirus	Murine xenograft of human pancreatic cancer	81
		Three-fold increased circulation time, reduced immune neutralization of OV and significantly reduced tumor growth	
Methoxy PEG-b-poly{N-[N-(2-aminoethyl)-2-aminoethyl]-l-glutamate} + PEG	Oncolytic adenovirus	Murine xenograft of human fibrosarcoma and lung cancer	83
		Increased accumulation in the tumor, reduced OV accumulation in off-target tissues, tumor growth reduced two- to three-fold	
Polymeric micelle + PEG	Oncolytic adenovirus, paclitaxel	Murine xenograft of human breast cancer	84
		Higher viral replication in tumor, 12-fold increased blood retention and 2.5-fold anti-tumor efficacy. Synergistic effect of paclitaxel + OV	
Chitosan–PEG folate	Oncolytic adenovirus	Murine xenograft of human epithelial cancer	85
		Increased blood circulation time, two-fold reduced tumor growth	
Lipid nanoparticle	mRNA cancer vaccine	Mouse melanoma and colon cancer models	86
		Completely inhibited tumor growth	
		Clinical trial for malignant melanoma: *De novo* T cell responses against the cancer	

Liposomic nanoparticles conjugated to stimulatory factors such as IL15/IL21 allowed complete tumor elimination, compared to limited survival increase with ACT and systemic stimulatory factors without nanoparticles.^77^ PEG–PLGA nanoparticle-conjugated PD-1, combined with antitumor necrosis factor receptor superfamily member 4, ensured simultaneous binding to the two molecules and showed a tumor-free survival rate of 30% after biomaterial treatment, compared to 10% after treatment with a mix of NP-conjugated and free drugs in a murine B16F10 melanoma tumor model.^78^ Nanoparticles also offer interesting possibilities for the treatment of lung cancer: inhaled cationic chitosan nanoparticles conjugated to anti-PD-L1 were able to adhere to the lung mucus layer to prolong anti-PD-L1 retention as well as act as an immune adjuvant due to the inherent immunostimulatory qualities of chitosan.^79^ Increased mouse survival and fewer metastases were seen in a mouse model of B16-F10 lung metastases as compared to free drugs or nanoparticles alone.

Nanoparticles have been utilized as synthetic APCs, such as a study where iron-dextran and quantum dot nanoparticles were conjugated to CD28 and major histocompatibility complex (MHC)–peptide complexes to form synthetic APC, leading to a significant reduction in tumor growth in mice.^80^

Some groups have conjugated nanoparticles with OV, to prolong their circulation time and improve their antitumor efficacy. Brugada-Vilà *et al.* developed an OV conjugated with PEGylated oligopeptide-modified poly(β-aminoester)s that showed a three-fold increased circulation time, reduced immune neutralization of OV, and significantly reduced tumor growth in murine xenograft models compared to unconjugated OV using the human pancreatic cell lines PANC-1 or MIA PaCa-2.^81^ Methoxy poly(ethylene glycol)-b-poly{N-[N-(2-aminoethyl)-2-aminoethyl]-l-glutamate} and PEG grafted to the OV increased accumulation in the tumor and reduced OV accumulation in off-target tissues.^82^ Tumor growth was also reduced two- to three-fold compared to unconjugated OV in murine xenograft models using HT1080 human fibrosarcoma and A549 lung cancer cell lines.^83^ OV + paclitaxel conjugated with PEG and a polymeric micelle demonstrated higher viral replication in tumor, 12-fold increased blood retention and 2.5-fold increased anti-tumor efficacy compared to unconjugated OV in murine xenograft models using the human breast cancer cell line MCF-7 compared to free OV + PTX.^84^ Chitosan PEG folate nanoparticles greatly increased blood circulation time and showed a two-fold reduced tumor growth compared to unconjugated OV in murine xenograft models using the KB human epithelial carcinoma cell line.^85^

Nanoparticles have also been used in cancer vaccines, interestingly including mRNA delivery as the vaccine technology. Lipid nanoparticles for mRNA vaccine delivery showed potent anti-tumor effects in mice and some success in clinical trials to treat melanoma.^86^ In this case, mRNA coding for cancer antigens was encapsulated in negatively charged lipid nanoparticles, which protect the mRNA from elimination by the immune system and efficiently transport the mRNA to APC in the spleen. The mRNA expression appears to mimic infection with RNA viruses where free DNA and RNA are sensed by APC via receptors such as TLR7, which in turn stimulates the production of IFN-α that activates DC, NK, B, and T cells. Furthermore, the RNA is internalized and translated by DC resident in the spleen, producing antigens that are then presented by the DC to activate and stimulate the proliferation of antigen-specific T cells. This technology was also one of the precursors to the revolutionary and now ubiquitous mRNA vaccines developed by Pfizer and Moderna against COVID-19.^87^

### C. Scaffolds for immune cell delivery

#### 1. Principles of ACT

Another therapeutic strategy for which biomaterials could bring strong benefit is ACT. Conventional ACT consists of the intravenous administration of immune cells, mostly of T lymphocytes, which are expanded *ex vivo* and implanted into the patient to harness their specific anti-tumor effects. Presently, there are two main types of ACT treatment, depending on the sources of immune cells: tumor infiltrating lymphocytes (TILs) and genetically modified T cells with chimeric antigen receptors (CARs) or with tumor-specific TCRs.

**FIG. 8.**
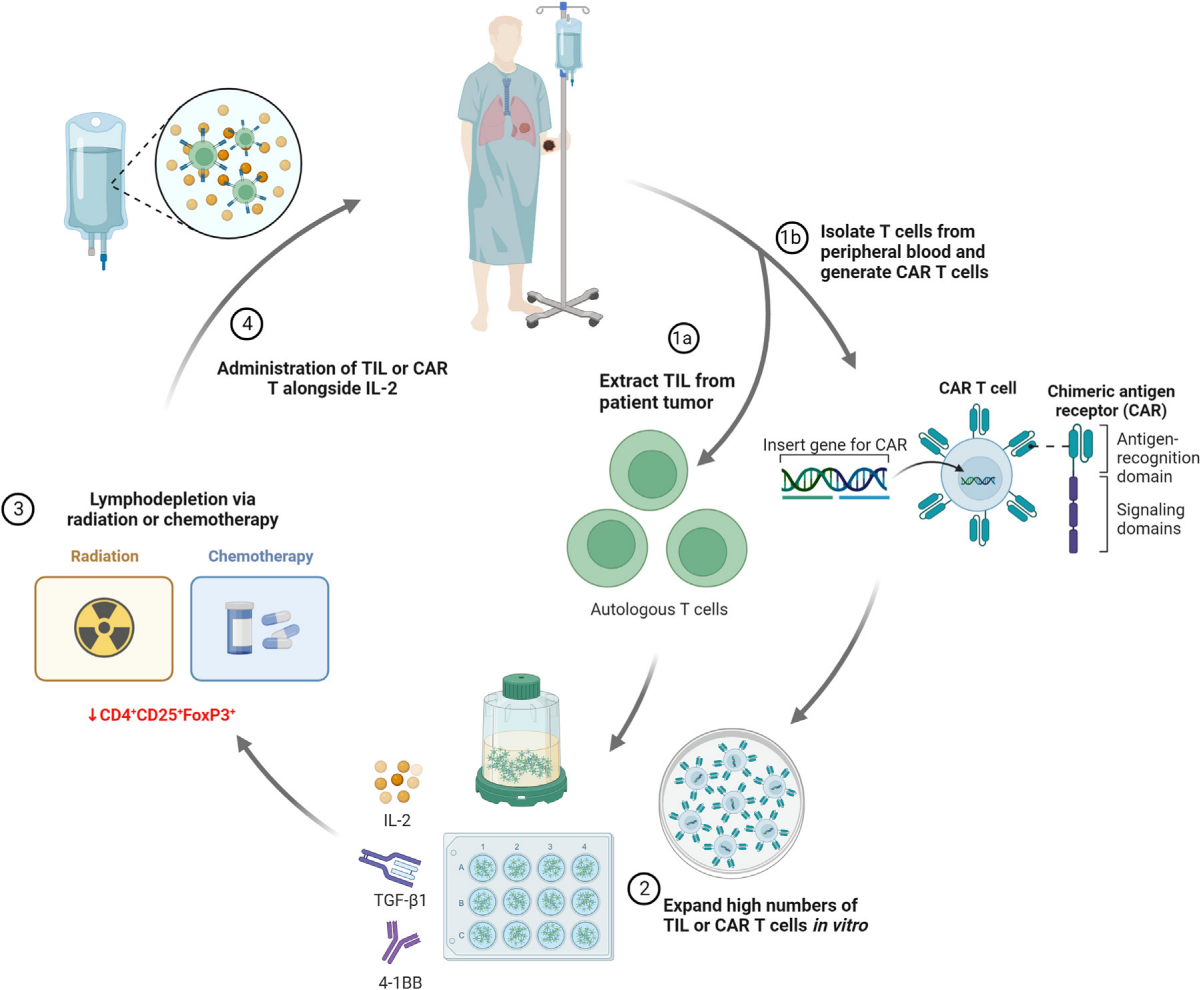
Typical phases of in clinical ACT: TIL or CAR are obtained and expanded *in vitro*, before lymphodepletion and re-administration alongside growth factors, such as high- or low-dose IL-2. CD4^+^ CD25^+^ FoxPE^+^ cells are immunosuppressive regulatory T cells that downregulated the activity of cytotoxic T lymphocytes in ACT.

TIL used are obtained from the patient tumor, due to their well-established, inherent tumor-specific response, considerably more abundant than peripheral lymphocytes. Since its first adoption in 1988 by the team of Rosenberg *et al.*, TIL-ACT has shown great promise in the treatment of metastatic melanoma. In this treatment, TIL must be isolated from the tumor and expanded *in vitro* to reach 1–20 × 10^9^ cells, before being intravenously reinjected in the patient. IL-2 is an essential growth factor for T cell proliferation, though other growth factors, such as 4–1BB and TGF-β1, have been proposed as additional factors to improve cell growth and the anti-tumor response.^88,89^ Current ACT with TIL also uses a rapid expansion protocol, where minimally cultured and non-selected “young” TIL have been shown to be more effective than TIL cultured over a longer time period and selected *in vitro* based on antigenic stimulation,^11^ though a limited number of treatment centers are able to both rapidly culture young TIL and select them for tumor specificity through the identification of unique tumor mutations via whole exome sequencing.^90^ Lymphodepletion via a combination of chemotherapy and total body radiation, prior to T cell injection, is also considered to have a beneficial effect on the anti-tumor activity. High dose IL-2 is also administered in order to maintain T cell activation *in vivo*, which is a source of the toxicity and negative side effects of current ACT.^91^ The main steps of ACT are summarized in Fig. 8.

Another large area of interest is in so-called CAR-T cells, where T cells are genetically modified to express CARs in addition to the native T cell receptors. Their advantage lies in the fact that their recognition is not MHC-dependent, and they can recognize a wider variety of antigens than T cell receptors (which are limited to short peptides). Several pitfalls, however, keep these promising treatments from becoming mainstream.

One significant limitation of ACT is the large numbers of T cells that must be obtained, as many are lost to non-cancerous sites of inflammation during systemic administration and only a small fraction is reaching the tumor. This expansion can be lengthy, expensive, and difficult to achieve for some patients, causing many to lose treatment eligibility because of their deteriorating condition. Cells can also struggle to persist and survive in the immunosuppressive tumor microenvironment.^92–94^ Even CAR-T cells, despite their success, show similar problems to ACT with TIL, including toxicities, such as cytokine release syndrome and neurotoxicity, as well as limited efficacy in solid tumors.^95^

These limitations call for reduced dosage and local cell delivery, in order to reduce the number of cells needed and avoid IL-2 associated toxicity, resulting in safer treatments. Simple cell injection around tumors is not sufficient, due to rapid cell loss due to dispersion, inflammation, and anoikis.^96^ Biomaterial scaffolds could increase the number of cells at the target tumor site, while decreasing the overall doses and numbers required.^58^ Furthermore, the inclusion of drugs or molecules to improve T cell persistence and anti-tumor activity, as well as the improved physical retention of T cells at the tumor site when delivered via a biomaterial, could improve cell persistence and functionality as well.

#### 2. Design criteria for immune cell scaffolds

Cell-delivery scaffolds refer to biomaterials used as structures with defined architecture and composition, used for the delivery, retention, and support of cells, possibly combined with therapeutic molecules. Only recently have cell-delivery scaffolds been proposed to enhance the efficacy and overcome the limitations of ACT treatments. In terms of the requirements for an immune cell delivery scaffold, the scaffold should satisfy several criteria:^18,97^

1. Ease of administration—the scaffold must ideally be capable of minimally invasive implantation, via either a catheter or injection of the scaffold through a needle, to avoid the complications of surgical insertion and reach any location within the body.
2. Easy and simple homogenous mixing with cells, with a matrix capable of cell protection from applied shear stress during injection through standard needles or catheters.
3. Rapid stability *in vivo*—after minimally invasive implantation the scaffold must form a mechanically stable structure, stationary in the location it has been delivered in the body to ensure cell retention close to the target site.
4. Cell compatibility—in addition to the general biocompatibility of the scaffold, it must support cell encapsulation. Once *in situ* the scaffold must support the survival, growth, anticancer function, and escape of immune cells over a timeframe sufficient for cancer treatment.
5. Porosity—the scaffold must have a porosity that allows access to nutrients and waste removal for encapsulated cells and allows immune cells to escape and perform their anti-cancer functionality, eliminating cancerous cells while other immune cells, such as DC and B cells, could colonize scaffolds to form protective tertiary lymphoid tissue.
6. Biodegradability—the scaffold should biodegrade into nontoxic, metabolizable products. In the case of cancer immunotherapy, degradability such that when the tumor is eliminated the biomaterial will also degrade and disappear would be desirable.
7. Formulation—the scaffold should ideally be chemically simple to formulate and avoid toxic chemical processes and to ease fabrication and approval by regulatory bodies, such as the FDA in the USA or the European Medicines Agency in Europe.
8. Sterilizability—the biomaterial should be capable of sterilization using standard methods, such as autoclaving, ethylene oxide, or gamma radiation, prior to cell mixing and injection.

**FIG. 9.**
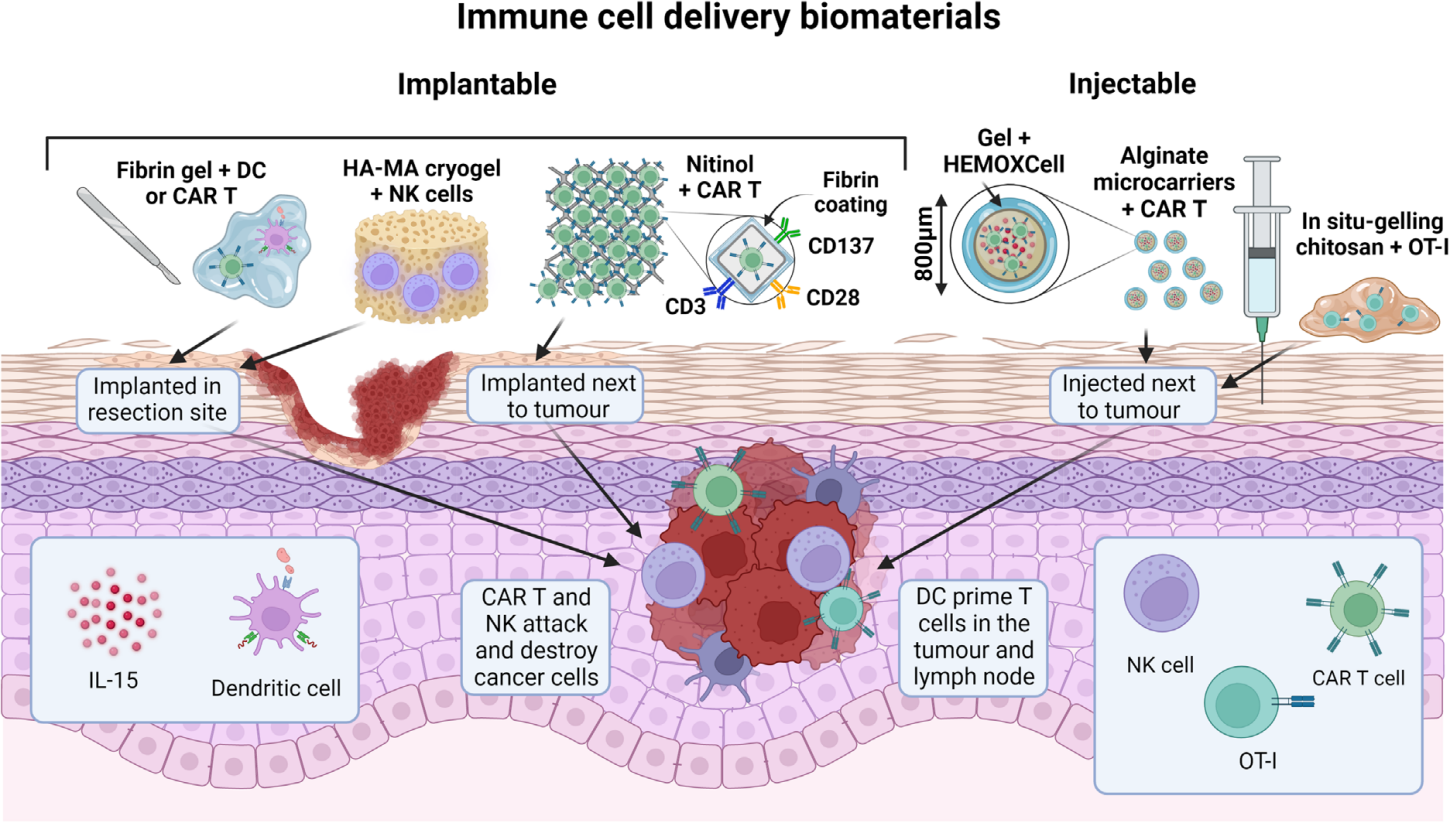
Selected biomaterials for immune cell delivery. Implantable or injectable gels, meshes, and microcarriers are loaded with dendritic cells, NK cells, or CAR T cells to improve cell delivery and persistence in and around the tumor. Cells progressively escape from the scaffolds toward the tumor and immune system, while the biomaterials and additions, such as stimulatory antibodies or cytokines, augment treatment efficacy.

Table V gives a summary of recent or current studies of cell delivery scaffolds for cancer immunotherapy, which run from simple *in vitro* studies to preclinical murine models and use lymphocytes, DC, and even in one case NK cells. These are detailed further in Sections C3 and 4 a selection are shown in Fig. 9.

**TABLE V.** Cell-delivery biomaterial scaffolds for immunotherapies.

Materials	Cells	Therapeutic agents	Injectable?	Immunotherapy model and outcomes	Reference
Chitosan–PEG	Human PBMC	None	Yes	*In vitro* glioblastoma cells	98
Chitosan–PEG	Human CAR-T cells	Self-expression of IL-15	Yes	Human retinoblastoma	99
				Elimination of tumors and 100% survival with gel-delivered IL-15 CAR	
PCL–PEG–PPG copolymer	Human CD4^+^ T cells	None	Yes	Human CD4^+^ T cell survival over 5 days	100
PEG–heparin	Human CD4^+^ T cells	CCL21	Yes	Increased human CD4^+^ T cell proliferation	101
PEG	Human CD3^+^ T cells	None	Yes	Proliferation upon restimulation of escaped T cells	102
Chitosan	Human PBMC and TIL	None	Yes	*In vitro* renal cancer, breast cancer and melanoma	103
Polyisocyanopeptide (PIC) + GRGDS peptide	Human T cells, DC and NK, mouse T cells	None	Yes	Migration in mice *in vivo* without tumors	104
Hyaluronic acid	Human CAR-T cells	None	Yes	*In vitro* glioma cell line	105
Alginate + GFOGER collagen-like peptide	Human CAR-T cells	IL-15 agonist, CD137, CD28, CD3	No	Mouse breast cancer resection model	106
				Regression in 60% of treated mice vs 0% survival in untreated mice	
Alginate + GFOGER collagen-like peptide	Human CAR-T cells	IL-15 agonist, CD137, CD28, CD3, STING agonist	No	Mouse pancreatic cancer and melanoma models	107
				Complete pancreatic tumor elimination in 40% of treated mice, with persistent immunity on tumor rechallenge.	
Fibrin-coated Nitinol	Human CAR-T cells	IL-15 agonist, CD137, CD28, CD3	No	Mouse ovarian cancer model	108
				2.7-fold survival increase with CAR-T-loaded Nitinol film vs untreated	
Fibrin	Murine DC	None	Yes	Mouse lung cancer model	109
				Encapsulated DC treatment more effective than non-encapsulated	
RADA16 peptide	Murine DC	Tumor antigens, anti-PD-1	No	Mouse lymphoma model	110
				Survival improvement with gel-encapsulated DC vs untreated mice	
α-CD/PEG	Murine DC	DOX, CpG, B16 tumor cells	Yes	Mouse melanoma model	111,112
				Significant survival improvement with gel-encapsulated DC with CpG + B16 vs single treatments	
Gelatin-hydroxyphenyl propionic acid	Murine DC	OV	Yes	Mouse lung cancer model	113
				Increased survival compared to single treatments of DC/OV	
Hyaluronic acid	Human CAR NK cells	None	No	Mouse leukemia and breast cancer models	114
				Significant survival improvement with gel-encapsulated CAR NK vs non-encapsulated NK	
Alginate microspheres	Human CAR-T cells	IL-15, hemoglobin	Yes	Mouse renal and ovarian cancer models	115
				Greatly improved mouse survival with alginate-encapsulated CAR-T cells compared to untreated mice	
Fibrin	Human CAR-T cells	None	No	Mouse lymphoma and glioma models	116
				Significantly improved mouse survival with fibrin-encapsulated CAR-T cells compared to mice treated with non-encapsulated CAR-T cells	
Methacrylated hyaluronic acid	Human CAR-T cells	IL-15 nanoparticles, anti-PD-L1 platelets	No	Mouse model of human melanoma	117
				Extensive tumor elimination with CAR + IL-15 + anti-PD-L1	
Alginate	Monocytes	Tumor antigens, anti-PD-1	Yes	Mouse breast cancer modelSignificant preventative and therapeutic anti-tumor effects	118

#### 3. T lymphocyte-loaded scaffolds for ACT

Cell-delivery scaffolds offer improvements to systemic cell delivery using inherent biomaterial advantages, such as localization and the incorporation of immunostimulatory factors. T cell-delivery scaffolds have been the most studied approach, with a range of both implantable and injectable materials been investigated with varying progress from *in vitro* work to *in vivo* models.

##### a. Implantable scaffolds

Stephan’s group, which is prominent in this area, developed oxidized (and therefore biodegradable) alginate gels grafted with a collagen-mimetic peptide to locally deliver CAR-T cells.^106^ *In vitro* studies demonstrated the benefit of this adhesive peptide, which increased lymphocyte migration within the gel and lymphocyte escape into a surrounding collagen gel, as well as increasing cell viability compared to unmodified alginate. Lipid-coated mesoporous silica microspheres (MSM) incorporating an IL-15 agonist as well as the immunostimulatory antibodies CD3, CD28, and CD137, similar to those present in the Dynabeads Human T-Activator commonly used for *in vitro* T cell expansion, were also included, and the scaffold was lyophilized to create an implantable porous matrix that was seeded with T cells immediately before implantation. The scaffold led to very promising *in vitro* and *in vivo* results in mouse models. In breast cancer resection model, it reduced tumor relapse compared to conventional intravenous or peritoneal treatments and supported tumor-targeting T cells throughout resection beds and associated lymph nodes, while it triggered much stronger regression in a multifocal ovarian cancer model resulting in greater mouse survival than locally delivered cells without the scaffold.

The same group further developed this model in 2017, adding cyclic di-GMP as a STING agonist, again loaded into the MSM, to activate DC and further enhance the immune response.^107^ The combination showed increased efficacy in treating solid pancreatic cancer and melanoma tumors in mice, with the STING agonist addition resulting in complete tumor remission in some cases. The authors conclude that the codelivery of STING agonists can stimulate the immune responses to eliminate tumor cells that are not recognized by the adoptively transferred lymphocytes and thus improve the CAR-T cell therapy and help protect against the emergence of escape variants. However, the scaffold is not injectable and its numerous complicated fabrication steps were a strong limitation toward clinical transfer. More importantly it lacked a well controlled porosity to favor T cell survival and proliferation.

More recently, Stephan's group adapted similar modifications to a fibrin-coated nickel–titanium alloy (nitinol) porous mesh with well controlled porosity, that can be placed on tumor lesions and release and functionally support tumor-targeted T cells.^108^ These micropatterned thin films (approximately 10 *μ*m), formed via magnetron sputtering, were designed to improve oxygen and nutrient transfer to the T cells which was a limiting factor in their previous alginate gels. Nitinol is inherently bioinert due to the thin layer of titanium oxide formed on its surface, though in this work the nitinol was functionalized through fibrin coating which allowed lymphocyte adhesion and migration as well as coupling to the CD3, CD28, and CD137 antibodies. The system elicited robust proliferation of the seeded T cells *in vitro* and *in vivo*, leading to a huge increase in T cell on the site compared to intravenous and locally injected cells. The authors also showed impressively enhanced tumor elimination compared to locally or intravenously injected CAR-T cells.

The necessity to implant this scaffold is still a potential limitation compared to injectable cell delivery scaffolds for immunotherapy. However, the mesh can be incorporated into a variety of implant configurations, such as an endovascular stent for minimally invasive administration. Applicability to human patients may depend on tumor location and feasibility of surgical implantation. Moreover, the practical constraints for producing and manipulating sterile nitinol films, and their subsequent seeding with T cells followed by implantation or catheter administration may be limiting for practical clinical use.

**FIG. 10.**
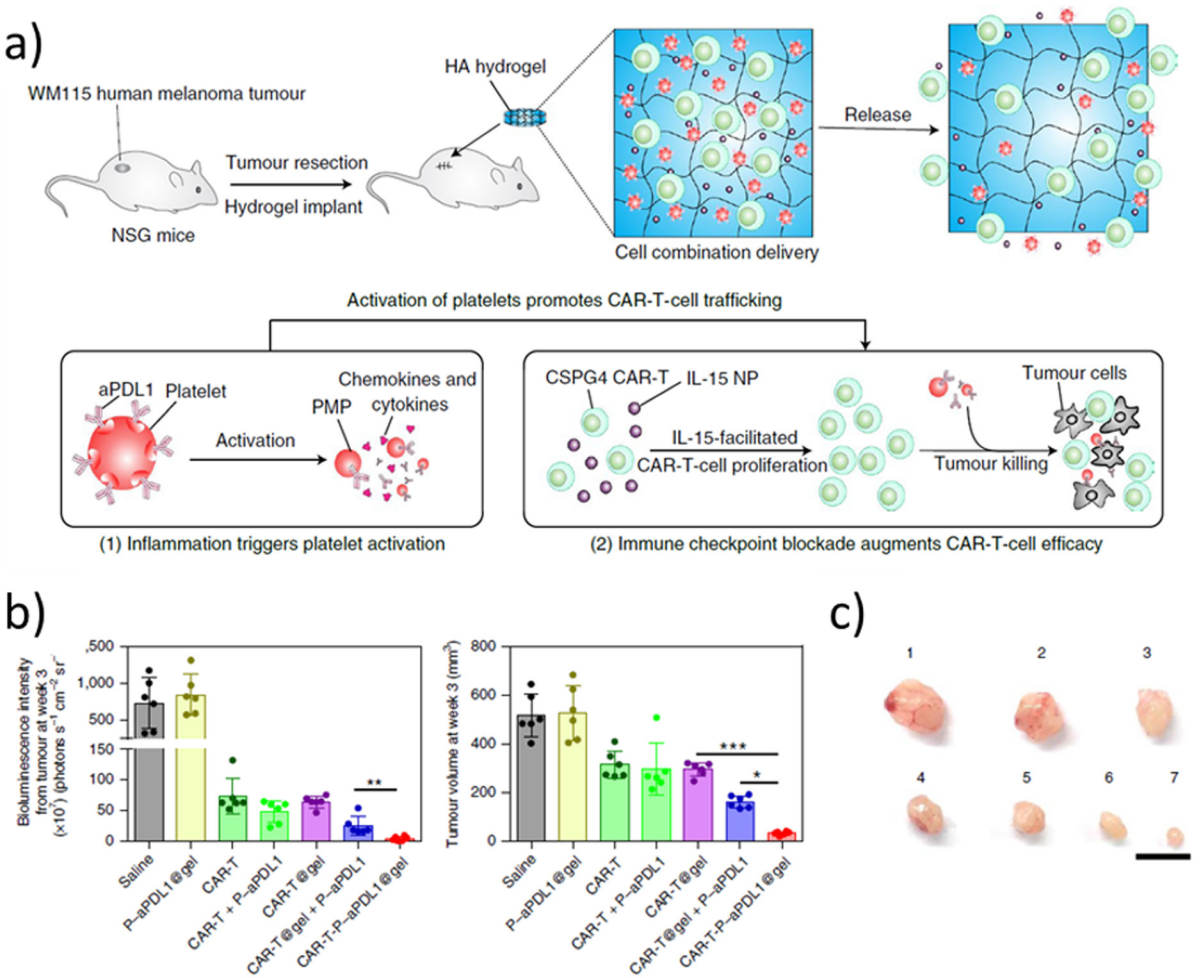
Cryogel for CAR-T cell delivery. (a) Schema of the tumor model and cryogel. (b) Tumor bioluminescence (left) and volume (right) 3 weeks after treatment (mean ± SD, n = 6 mice per group). Statistical analysis was performed using one-way ANOVA followed by Tukey's HSD post-hoc test. (c) Representative photos of tumors at three weeks. Groups: (1) saline; 2, P-aPDL1@gel; (3) CAR-T; (4) CAR-T + P-aPDL1; (5) CAR-T@gel; (6) CAR-T@gel + P-aPDL1; and (7) CAR-T-P-aPDL1. Scale bar, 1 cm. PMP: platelet-derived microparticles, P-aPDL1: PDL1 antibody covalently conjugated to the cell surface of human platelets. Reproduced with permission from Hu *et al.*, Nat. Biomed. Eng. **5**, 1038–1047 (2021). Copyright 2021 Springer Nature.

Another interesting study is a methacrylated hyaluronic acid hydrogel developed by Gu’s group for CAR-T cell encapsulation, using freeze-drying to create implantable porous hydrogels with a similar method to the previously described cryogel vaccines.^117^ The gel also incorporated IL-15 loaded PLGA nanoparticles, and platelets conjugated to anti-PD-L1. While the effect of the CAR-T-loaded hydrogel was quite limited, almost complete tumor elimination was achieved with the group with co-encapsulated CAR, IL-15, and PD-L1 in a mouse model using WM115 human melanoma cells [Fig. 10(b)]. They also demonstrated by bioluminescence the persistence and growth of T cells *in vivo*, and the scaffold triggered an abscopal effect inhibiting distal tumor growth.

##### b. Injectable scaffolds

Several efforts have also been made to create injectable T lymphocyte delivery scaffolds. A thermosensitive chitosan–PEG hydrogel, prepared by alkylation of chitosan followed by Schiff base formation, showing loading, survival, escape and anti-cancer activity of T lymphocytes *in vitro* against a glioblastoma cell line.^98^ However, the scaffold had a gelation time of 8–12 min at 37 °C, which is quite long to avoid cell dispersion at the time of *in vivo* injection. The same gel was later applied to CAR-T cells targeted against GD2, an antigen overexpressed in retinoblastoma.^99^ The gel prolonged CAR persistence within the tumor and gel-encapsulated CAR significantly reduced tumor growth. Impressively, further modification of the CAR to induce self-expression of IL-15 resulted in tumor elimination and a 100% survival rate. Our group also developed a thermosensitive porous chitosan gel for T cell delivery, demonstrating significantly faster gelation at 37 °C than the chitosan–PEG gel and human PBMC viability and growth over 2 weeks, as well as specific anti-cancer functionality in transwell models.^103^ This physical gel requires no chemical modification or cross-linking of chitosan, a natural biomaterial already used in FDA-approved medical devices. It is completely biodegradable and can be prepared by simple mixing of two sterilized solutions following by the addition of the cells prior to injection through small diameter syringes (up to 23G). These are important advantages for the potential clinical transfer of such a cell delivery scaffold for immunotherapy. *In vivo* tests in MC38-OVA murine subcutaneous tumor models have shown some efficacy to reduce tumor growth *in vivo*, even when administering a reduced cell number compared to systemic treatment.^119^ However, the effect is limited in time and the absence of cell proliferation inside the scaffold *in vivo* calls for further gel optimization.

Perez del Rio *et al.* developed a PEG-heparin hydrogel containing lymphocyts,^101^ where heparin allowed the conjugation of the cytokine CCL21 which is present in the lymph nodes and is known to enhance T cell proliferation and migration.^120^ The hydrogels increased CD4^+^ T cell proliferation, though were demonstrated primarily as T cell culture scaffolds rather than a delivery platform. A PCL–PEG–poly(propylene glycol) copolymer has also been demonstrated for T cell encapsulation, though only showed cell survival over 5 days and no proliferation was demonstrated in this case.^100^ Yan *et al.* demonstrated a PEG hydrogel formed via Diels–Alder cycloaddition of fulvene and maleimide functionalized PEG precursors, which has tunable stiffness and degradation.^102^ However, the gel showed relatively slow gelation (>15 min) and limited cell viability after several days even with RGD functionalization, though cells escaped from the gel showed proliferation after recovery and re-stimulation.

In the Netherlands, Figdor's group investigated RGD grafted polyisocyanopeptide (PIC) gels to culture human T cells and observe the *in vivo* escape of murine T cells.^104^ PIC, a relatively new class of hydrogel, are synthetic hydrogels formed through nickel(II)-catalyzed polymerization of the two monomers—triethylene glycol functionalized isocyano-(D)-alanyl-(L)-alanine (monomer 1) and monomer 2, an azide-appended version of monomer 1. This water-soluble polymer exhibits thermosensitive gelation, remaining liquid below 16 °C and forming soft hydrogels in a matter of minutes above this temperature.^121^ Interestingly, its mechanical properties can be tuned to reach similar mechanical properties and stress-stiffening behavior to biological polymers such as collagen.^122^ The authors showed that GRGDS peptide grafting allowed greater T cell migration within the gel compared to unmodified gel and *in vitro* viability and proliferation up to 72 h.^104^ Scaffold–encapsulated T cells migrated out of the gels over 3–4 weeks in mice, though with many T cells escaping the gel in the first week after injection. Furthermore, the study was performed simply to demonstrate T cell escape from the gel in mice without tumors and so has not demonstrated efficacy in a mouse tumor model. Atik *et al.* developed a proprietary, low-viscosity hyaluronic acid and gelatin-based gel as a substrate to deliver CAR-T for glioblastoma using convection enhanced delivery, in which an intracranial catheter is placed into the tumor that infuses the agent with positive-pressure over time.^105^ The encapsulated CAR-T cells migrate from the gel and carry out their cytotoxic function *in vitro*. The gel itself showed no toxicity when injected in mice, though again this was performed in mice without tumors and so has not demonstrated anti-tumor efficacy.

Other groups are also beginning to investigate alternative materials and biomaterial formats for ACT delivery. Fibrin gel-mediated CAR-T cell delivery was, thus, tested for glioblastoma by inoculating fibrin solution with CAR-T cells in the cavity followed by immediate addition of thrombin solution. However such a method does not ensure complete encapsulation due to cell dispersion prior to gel formation and fibrin gel is known to degrade very rapidly.^116^

The main challenge with injectable scaffolds is to create macroporous structures which allow good access to oxygen and nutrients and the possibility for the cells to escape the scaffold. Several studies including Stephan's work with nitinol films have shown that the small physical size of microscaffolds is beneficial for nutrient supply and cell migration.^108,123^ Another alternative is to encapsulate T cells in small microspheres that allow better diffusion of oxygen and nutrient, and potential vascularization in between the microspheres.^124^ Thus, Luo *et al.* created an injectable hydrogel-encapsulated porous immune-microchip system (i-G/MC) with the capabilities of enhancing CAR-T cell survival and proliferation.^115^ Interestingly, they incorporated the HEMOXCell molecule in their alginate microspheres, a marine hemoglobin with a high oxygen storage capacity, to counteract the hypoxic effect of the tumor microenvironment. However, the long and complex preparation steps required (including multiple lyophilisations and several days immersion in PBS) may prevent the use of such systems in clinical trials.

Altogether, despite impressive developments and a variety of approaches, and very promising results in mice models, there is presently no ideal scaffold for T cell delivery which would combine well controlled porosity and injectability and clinically feasible manufacturing processes. Moreover, efficacy against distal or metastatic tumors and long-term tumor elimination will ideally need to be demonstrated for eventual progression to clinical trials.

In several cases, the authors demonstrate further increase in mice survival when adding debris from eradicated tumor cells to convert the implant to not only act as a cell delivery but also a “self” vaccine site. Thus, to mount a robust anticancer response, an ideal scaffold should probably not only allow sustained growth and delivery of a sufficient number of stimulated T cells that destroy tumor cells but also contain significant amounts of tumor antigens which become available to endogenous APCs and eventually high concentrations of stimulants that activate these APCs.

#### 4. Scaffolds for other immune cells

Biomaterials have also shown promise to assist DC-based immunotherapies, with DC probably the most commonly delivered immune cell via biomaterial scaffolds after T cells. Such DC loaded scaffolds can overcome the lack of recruitment of host DCs to create more efficient vaccine-like immune cell niches. DC can be stimulated prior to their addition in the gel or injected in combination with the tumor antigen, and eventually other drugs, as detailed below.

Tumor antigen-stimulated DCs in a fibrin gel showed significantly reduced tumor growth in mice with the cell scaffold construct compared to injected DC alone.^109^ DC were also delivered using an injectable self-assembled peptide hydrogel,^110^ based on RADA16, a synthetic peptide consisting of 16 alternating hydrophobic and hydrophilic amino acids which self-assembles into a nanofibrous, nanoporous hydrogel in the presence of neutral pH solution.^125^ DC and antigens were mixed with the hydrogel on ice before injection. The gel was biocompatible with DC, did not activate DC by itself (being non-immunogenic) and when containing DC and antigens it improved both therapeutic and prophylactic efficacy in reducing tumor growth in a mouse lymphoma model, compared to intravenous and subcutaneous injection, with even greater efficacy observed in conjunction with an anti-PD-1 checkpoint inhibitor. In addition to anti-PD-1, Yang *et al.* showed that the chemotherapeutic DOX in the form of nanoparticles, also potentially conjugated to the immune adjuvant CpG, could be incorporated in an injectable α-cyclodextrin/PEG hydrogel along with DC. The gel significantly reduced tumor growth in a B16 melanoma mouse model compared to single treatment or control groups when treated with the full complement of DC, DOX and CpG-loaded gel.^111^ Subsequent work from this group also showed the further beneficial effect of including dying B16 tumor cells into the DC scaffold vaccine.^112^ A similar trend was also observed with monocytes encapsulated in alginate droplets using a microfluidic system.^118^

The combination of IL-12 and GM–CSF-expressing OV with DC was tested using a gelatin-hydroxyphenyl propionic acid hydrogel, enzymatically cross-linked via horseradish peroxidase (HRP) and hydrogen peroxide (H_2_O_2_). Gel-encapsulated OV + DC significantly increased survival in a murine Lewis lung carcinoma model compared to single treatments of a DC/OV combination without gel, with impressive 100% survival in mice treated with Gel OV + DC. All this work indicates the range of materials and drugs beneficial for monocyte and DC delivery, as with T cell ACT.

Another interesting recent development is a hyaluronic acid-based scaffold that was used for the delivery of NK cells as a cancer immunotherapy. The scaffold was formed from a blend of methacrylate-modified HA and methacrylate-modified oxidized HA, where the methacrylate-modified oxidized HA acted as a highly degradable sacrificial component to create greater porosity and allow NK cell clustering which improves cell activation and viability.^126^ The scaffold upregulated NK cell proliferation and tumor killing activity *in vitro* and resulted in fewer metastases and increased mouse survival *in vivo.*^114^ This work may well be the first of many investigations into NK cell-carrier biomaterials as cancer immunotherapies, though the scaffold is non-injectable and must be implanted so it would be interesting to examine some of the above injectable scaffolds as NK cell carriers.

## III. CONCLUSIONS AND PERSPECTIVES

In this review, we summarize how biomaterials can enhance cancer immunotherapies by addressing some of the challenges of these approaches, which have been at the forefront of pharmaceutical breakthroughs in recent years and will continue to be so. Through their localized action and controlled release of cells, cancer antigens, drugs, immunomodulatory molecules, or combinations thereof, biomaterials can overcome the problem of rapid cell and antigen dispersion, as well as toxicity related to systemic delivery, and allow the combination of several products, with or without exogenous cells to eliminate cancer more efficiently.

For cancer vaccines, biomaterials can address numerous limitations that have so far hindered their progress toward the clinic. Multiple studies indicate increased potency using biomaterial vaccines compared to free vaccine components intravenously or subcutaneously injected, one factor of which is likely the “immune niche” created in porous biomaterials. This environment encourages antigen uptake by DC, often in a space that is protected from the immunosuppressive tumor microenvironment to concurrently improve DC persistence. Furthermore, biomaterials allow the inclusion of immunostimulatory adjuvants or drugs, in addition to the antigens or tumor cell lysates, and the controlled release of these factors further increases their potency. Exogenous DC cells can also be added to ensure a greater number of activated DC cells, although this implies more complicated preparation steps and regulations. Such products will most probably be used in the future, at least to prevent cancer recurrence or fight metastasis. The main limitations at this stage are on one hand, the absence of ideal injectable porous scaffolds, and on the other, limited persistence and long-term efficacy, with a lack of clinical data.

Biomaterial scaffolds for drug delivery offer similar benefits in terms of controlled and localized release of therapeutics, which increases the potency of ICI and allows combination therapies, for instance with chemotherapies. Localized biomaterial delivery also allows reduced dosages and hence a reduction in the toxicity associated with both chemotherapies and immunotherapies. Furthermore, innovative carriers, such as the sprayable gel, theranostic optic fibers, and inhaled nanoparticles, expand the range of possible methods to administer treatments.

While scaffolds for T lymphocyte growth and delivery remain a significant and underexplored research area, they are potentially advantageous compared to systemic lymphocyte delivery to reduce the numbers of required cells and decrease cell loss. Furthermore, their flexibility in incorporating a range of stimulatory factors or drugs, such as ICI, to better prime the cells and help them avoid cancer immune escape shows great promise for current and future treatments. Certain commonalities between the highest performing scaffolds, such as the inclusion of IL-15 superagonist, indicate priority molecules to include for successful treatment. One can also imagine the development of artificial tertiary lymphoid structures (TLSs) combining several immune cell types, as commonly seen around tumors and associated with a favorable prognosis.^127^ Other scientific developments, such as 3D printing and bioprinting, also offer hope for further improvements in therapy efficacy and control within immunotherapy, by creating complex implantable 3D structures with spatially defined cell organization. For example, Jin *et al.* showed improved T cell proliferation and reduced cell exhaustion induced by coaxial 3D printed alginate fibers.^128^ This could be beneficial in the creation of spatially defined artificial TLS with distinct stromal, T and B cell zones, previously attempted and reviewed elsewhere.^129,130^

There are, however, still several challenges. One of them is that delivered cells must access the lymphatic or vascular system for efficacy against metastatic tumors. Some groups have shown the presence of delivered cells in the blood and draining lymph nodes, though the mechanism of how exogenous cells reached the vascular system or lymph nodes is rarely investigated and could be examined in future work. The immunosuppressive microenvironment that limits intravenous ACT treatments seems to have been counteracted in many of these examples as well as in some of the discussed cancer vaccines. However, a challenge of immunotherapy linked to tumor immunosuppression is its limited applicability outside certain cancers. It would be interesting to see these localized treatments applied to xenografts of immune-resistant cancers as opposed to the cell lines generally used so far to see if their potency is retained.

Another challenge is the difficulty to satisfy all the requirements of cell growth, delivery, and persistence as well as optimal cell–cell contact and simple, easy to transfer technologies which are injectable, sterilizable and perhaps even with off-the-shelf capabilities. Quick, simple manipulation by clinicians is also of high importance.^131^ The highest performing T cell scaffolds discussed here show impressive cell encapsulation or seeding, cell growth, and potent anti-tumor effects, but their design may be too complex for clinical translation. A compromise may be necessary between efficacy and feasibility. The requirements for regulatory approval must also be considered if these biomaterial-aided treatments are ever to progress to regular clinical use.^132^ The vast majority of the immunotherapeutic treatments described above would follow the FDA approval pathway of a biologic drug as the primary mode of action of any immunotherapy is inherently biologic in nature. Therapies using cells or tissues would also have the designation of human cell, tissue, and cellular and tissue-based products with combinations of drugs and materials further classified as combination products. Biologic drugs must demonstrate safety, purity, and potency, while the biomaterial component of combination products must satisfy further criteria associated with the medical device approval pathway.^133^ In particular, the biomaterial scaffold must pass ISO 10993 biocompatibility requirement, where material cytotoxicity, sensitization, carcinogenicity, and degradability, among other parameters, are to be assessed.^134^ The device's potential for sterilization, mass production, and batch-to-batch variability should all be considered. Further considerations include the need for aseptic processing, transmissible disease testing, and traceability if cellular products are used as well as Good Laboratory Practice, Good Manufacturing Practice, and Good Clinical Practice throughout research and development, manufacturing, and clinical testing.^135^ All this requires a large extent of *in vitro* and *in vivo* pre-clinical models and clinical trials all demonstrating safety and efficacy for the approval of these products, although candidate treatments can benefit from a range of expedited programs, such as Fast Track, Breakthrough Therapy, and Accelerated Approval, where clinical or non-clinical data indicate a safety or efficacy improvement compared to existing treatments for serious conditions.^135^

As demonstrated along this review, biomaterials will likely be part of the future approaches to support and continue the revolutionary effect that immunotherapy has had on cancer treatment. As cancer immunotherapy is still at a relatively young stage in its development and scientific understanding, further progress in understanding cancer immunity and each sector of the immune system will guide future developments, such as the inclusion of ICI, adjuvants, and immunostimulatory factors with or without immune cells in biomaterial-based delivery systems. Cell delivery scaffolds represent a growing but still under-investigated area and may hold the key to more effective and durable future cancer treatments.

## Data Availability

Data sharing is not applicable to this article as no new data were created or analyzed in this study.
